# A New Perspective on the Pathophysiology of Idiopathic Intracranial Hypertension: Role of the Glia-Neuro-Vascular Interface

**DOI:** 10.3389/fnmol.2022.900057

**Published:** 2022-07-12

**Authors:** Per Kristian Eide, Hans-Arne Hansson

**Affiliations:** ^1^Department of Neurosurgery, Oslo University Hospital—Rikshospitalet, Oslo, Norway; ^2^Institute of Clinical Medicine, Faculty of Medicine, University of Oslo, Oslo, Norway; ^3^Institute of Biomedicine, University of Gothenburg, Göteborg, Sweden

**Keywords:** idiopathic intracranial hypertension, pseudotumor cerebri, benign intracranial hypertension, pathophysiology, intracranial pressure, astrocytes, capillaries

## Abstract

Idiopathic intracranial hypertension (IIH) is a neurological disease characterized by symptoms and signs of increased intracranial pressure (ICP) of unknown cause. Most attention has been given to the role of cerebrospinal fluid (CSF) disturbance and intracranial venous hypertension caused by sinus vein stenosis. We previously proposed that key pathophysiological processes take place within the brain at the glia-neuro-vascular interface. However, the relative importance of the proposed mechanisms in IIH disease remains unknown. Modern treatment regimens aim to reduce intracranial CSF and venous pressures, but a substantial proportion of patients experience lasting complaints. In 2010, the first author established a database for the prospective collection of information from individuals being assessed for IIH. The database incorporates clinical, imaging, physiological, and biological data, and information about treatment/outcome. This study retrieved information from the database, asking the following research questions: In IIH subjects responding to shunt surgery, what is the occurrence of signs of CSF disturbance, sinus vein stenosis, intracranial hypertension, and microscopic evidence of structural abnormalities at the glia-neuro-vascular interface? Secondarily, do semi-quantitative measures of abnormal ultrastructure at the glia-neurovascular differ between subjects with definite IIH and non-IIH (reference) subjects? The study included 13 patients with IIH who fulfilled the diagnostic criteria and who improved following shunt surgery, i.e., patients with definite IIH. Comparisons were done regarding magnetic resonance imaging (MRI) findings, pulsatile and static ICP scores, and immune-histochemistry microscopy. Among these 13 IIH subjects, 6/13 (46%) of patients presented with magnetic resonance imaging (MRI) signs of CSF disturbance (empty sella and/or distended perioptic subarachnoid spaces), 0/13 (0%) of patients with IIH had MRI signs of sinus vein stenosis, 13/13 (100%) of patients with IIH presented with abnormal preoperative pulsatile ICP [overnight mean ICP wave amplitude (MWA) above thresholds], 3/13 (23%) patients showed abnormal static ICP (overnight mean ICP above threshold), and 12/13 (92%) of patients with IIH showed abnormal structural changes at the glia-neuro-vascular interface. Comparisons of semi-quantitative structural variables between IIH and aged- and gender-matched reference (REF) subjects showed IIH abnormalities in glial cells, neurons, and capillaries. The present data suggest a key role of disease processes affecting the glia-neuro-vascular interface.

## Introduction

Idiopathic intracranial hypertension (IIH), also denoted pseudotumor cerebri (PTC) or benign intracranial hypertension (BIH), is a neurological disease characterized by symptoms and signs indicative of increased intracranial pressure (ICP), including headache, impaired vision, diplopia, and visual field findings of papilledema and reduced visual field, as well as dizziness, tinnitus, and fatigue (Ball and Clarke, 2006; Mollan et al., 2016). The prevalence of the disease is highest in fertile women who are overweight but may as well occur in children, normal-weight adults, and in men. Severe consequences are lasting visual impairment and blindness, and substantial negative effects on the quality of life. It is well-documented that the disease represents a great burden to those affected (Kleinschmidt et al., 2000). Patients with IIH are usually young and experience impaired occupational capacity which, also results in increased costs to society (Friesner et al., 2011). The occurrence of the disease is on the rise, probably related to the increasing weight in the population of the Western world (Kesler and Gadoth, 2001; Curry et al., 2005; Raoof et al., 2011; Kesler et al., 2014).

The pathophysiology of IIH is unknown (Mollan et al., 2016). Traditionally, most attention has been given to the role of cerebrospinal fluid (CSF) circulation failure, though the role of increased cerebral venous pressure caused by sinus vein obstruction has attracted interest more recently (Mollan et al., 2018). The current treatment options do not target the cause of disease, but focus on reducing ICP: Weight decline reduces abdominal pressure and thereby central venous pressure that may secondarily reduce ICP. Medications, e.g., acetazolamide and topiramate, reduce CSF production and CSF diversion surgery (shunt surgery) reduces CSF pressure by establishing an alternative route for CSF efflux. Given radiological evidence of sinus vein stenosis, stenting of dural veins, which lowers ICP by reducing back pressure caused by increased intracranial vein pressure proximal to the stenosis, has provided successful outcomes (Albuquerque et al., 2011; Kalyvas et al., 2020).

However, despite the best available treatments, a substantial proportion of the patients with IIH have lasting symptoms, a particularly disabling headache that extensively affects daily life (Friedman et al., 2017; Burkett and Ailani, 2018). Even mortality risk is increased in IIH (Hermes et al., 2020). One previous study estimated that annual costs for IIH in the USA in 2007 were 444 million USD, primarily related to frequent hospital admissions, unsatisfactory treatment options, and lost productivity (Friesner et al., 2011). The costs probably increase as weight is increasing in the population (Mollan et al., 2019). A 2015 Cochrane review concluded that there is a lack of evidence to guide medical or interventional treatment of IIH (Piper et al., 2015). Accordingly, there is a great need for a deeper knowledge of the pathophysiology of IIH for health care personnel to provide disease-specific treatment.

The first author since 2010 established a database (Neurovascular-Cerebrospinal fluid Quality registry, reg. no 2011/6692) that includes consecutive and prospective patients examined for IIH, as well as other patients with neurovascular and/or CSF disturbances. The database incorporates information from clinical management (clinical information, management data, and ICP measurements) and information obtained as part of research (analysis of brain biopsy specimens, intrathecal contrast-enhanced MRI assessing the glymphatic function).

**Figure 1:**
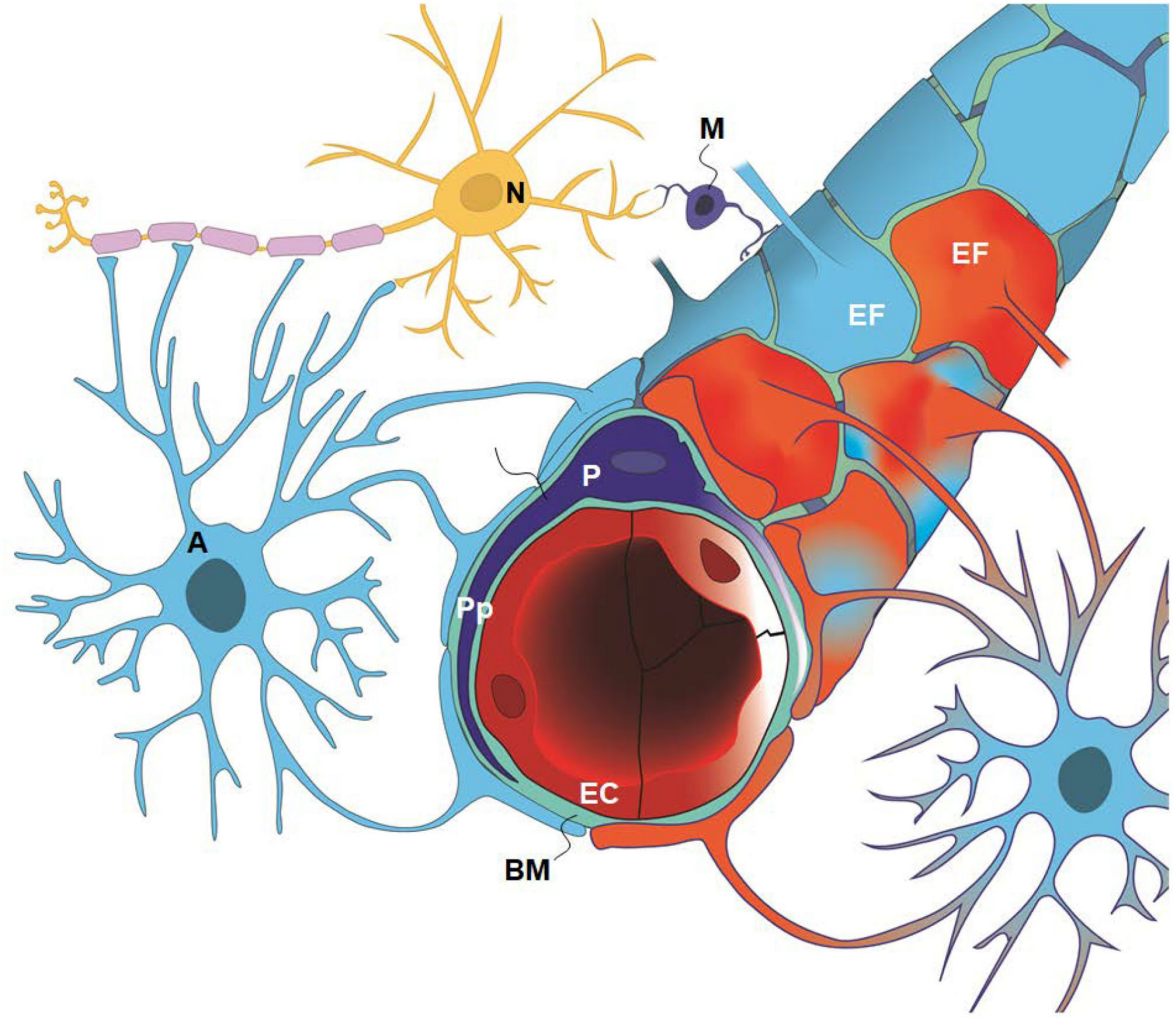
The glia-neuro-vascular interface (also denoted the glia-neuro-vascular unit/coupling) incorporates the capillary wherein its circumference consists of endothelial cells (ECs) having tight junctions and pericytes (P) with processes (Pp). The relationship between the endothelial cells and pericytes in humans is 1:1–3:1, decreasing with age and in diseases. The basement membrane (BM) is a loose matrix between endothelial cells (ECs) and pericytes (Ps) and the astrocytic end-feet (EF). The astrocytic end-feet processes create a donut shaped structure enclosing the basement membrane (BM). There are inter-end-feet-gaps between the endfoot (EF) processes enabling passage of molecules to the interstitial tissue. Normal end-feet (EF) are indicated in blue to the left while pathological end-feet (EF) in red color to the right. Other components are the astrocytes (A), neurons (N) and microglia (M). In IIH, there is patchy astrogliosis, causing changes to the normal configuration of astrocytes. Moreover, there is loss of integrity of the BBB with degeneration of basement membrane and pericyte processes, and BBB leakage of the blood proteins fibrinogen and fibrin. Extra-vascular fibrin(ogen) is not demonstrable in normal adult brains and is prominently pro-inflammatory. Our present disclosure of blood-brain-dysfunction in IIH subjects indicates that the glia-neuro-vascular unit is likely associated with the pathogenesis of the disease. Illustration: Ine Eriksen, University of Oslo.

The present study asked which of the variables indicative of IIH pathophysiology are most prevalent in IIH subjects who respond to CSF diversion surgery; specifically what is the occurrence of signs of CSF disturbance, sinus vein stenosis, intracranial hypertension, and ultrastructural abnormalities at the glia-neuro-vascular interface in patients with IIH? The main components of this anatomical location are glial cells (astrocytes and microglia), neurons pericytes, and, capillaries (Figure 1). This entity is also denoted as the neurovascular unit or coupling. Secondarily, a semi-quantitative measure of ultrastructural change was compared with a reference group from the same database. Study variables were retrieved from the already established database.

## Materials and Methods

### Database, Approvals, and Study Design

Since 2010, the Neurovascular-Cerebrospinal fluid quality control database has enrolled patients with neurovascular and CSF disturbances, including patients with IIH. The database has been approved by the Institutional Data Protection Official at Oslo university hospital (approval no. 2011/6692). The registry of patients with IIH stores prospectively collected information about the clinical status (preoperative symptoms and outcome results), imaging findings, overnight ICP scores, and results of ultrastructural analyses from cortical brain biopsies. The clinical information, overnight ICP recordings, and imaging data were included in the registry as part of routine clinical work-up in patients with IIH prior to shunt surgery. The morphological information about alterations at the glia-neuro-vascular interface was obtained as part of a research project approved by The Regional Committee for Medical and Health Research Ethics of Health Region South-East, Norway (Approvals no. REK 2009/2060, 2012/1157, and 2011/2306) and by Oslo University Hospital (Approvals no. 10/6806 and 2011/19311). The study was performed by the ethical standards as laid down in the 1964 Declaration of Helsinki and its later amendments, and patients were included after oral and written informed consent.

The design of the present study was to compare different variables addressing the pathophysiology of IIH, particularly measures of CSF disturbance, sinus vein stenosis, intracranial hypertension, and structural abnormalities from brain biopsy specimens. Data were retrieved from the prospectively collected database.

The primary research question was: In IIH subjects responding to shunt surgery, what is the occurrence of signs of CSF disturbance, sinus vein stenosis, intracranial hypertension, and microscopic evidence of structural changes at the glia-neuro-vascular interface?

The secondary research question was: Do semi-quantitative measures of abnormal ultrastructure at the glia-neurovascular differ between subjects with definite IIH and non-IIH (reference; REF) subjects, the latter individuals being matched with IIH subjects according to gender and age.

Inclusion criteria: (1) The patients with IIH included in the study fulfilled the IIH diagnostic criteria and had a definite clinical response to shunt surgery (i.e., improvement of visual function and at least partial improvement of headache). (2) The non-IIH (Reference; REF) subjects retrieved from the database were closest in age and gender to the IIH subjects. (3) This study was limited to IIH and REF subjects in the database who had analysis results of ultrastructural examinations and overnight preoperative ICP scores.

### Variables Retrieved From the Database

#### Demographic and Clinical Data

The demographic and clinical data retrieved from the database were age, gender, body mass index (BMI), preoperative symptoms, and management/outcome data. Moreover, they had undergone the routine management in this department, which is primarily placement of a ventriculoperitoneal (VP), or secondarily a lumboperitoneal (LP) shunt. For VP shunt, we apply HAKIM^TM^ Programmable Valve Shunt System (Codman & Shurtleff, Inc., Medos S.A. Rue Girardet 29, CH 2400 Le Locle, Switzerland), and for LP shunt we use an Orbis-Sigma Valve II shunt (Integra Inc, Plainsboro, NJ, USA).

The REF subjects closest in age to the patients with IIH were also retrieved from the Neurovascular-Cerebrospinal fluid database to compare ultrastructure at the glia-neuro-vascular interface. The database includes results from analysis of apparently normal brain tissue from individuals undergoing planned brain surgery in whom removal of normal brain tissue is required as part of treatment. This includes brain tissue resection for epilepsy, brain tissue resection during elective clipping of cerebral aneurysm (no prior bleeds) in whom minor tissue resection was required during the clipping procedure, and brain tissue from patients undergoing tissue resection for parenchymal brain tumors.

#### Imaging Signs of CSF Disturbance

**Figure 2:**
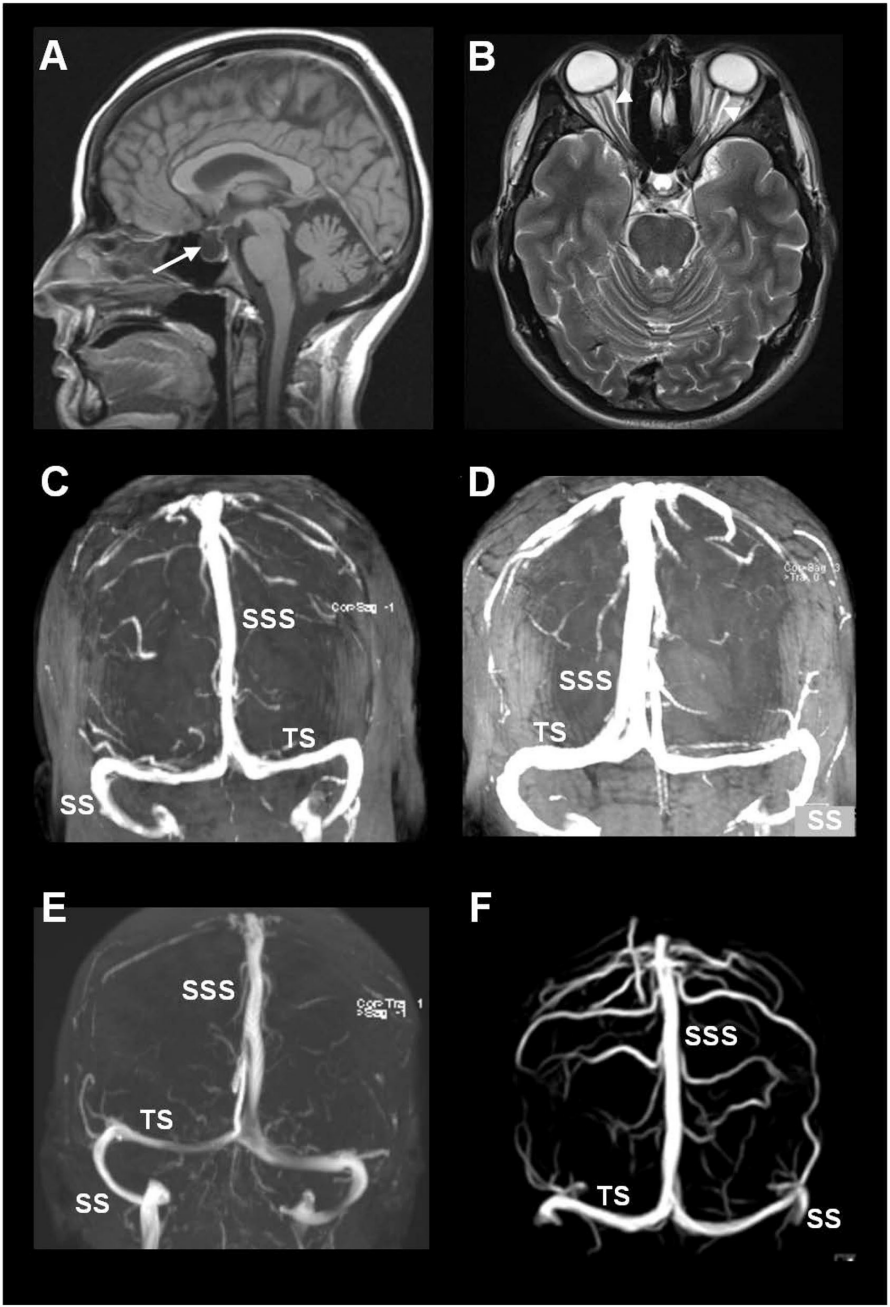
MRI biomarkers indicative of CSF disturbance in IIH include the presence of **(A)** empty sella (arrow) and **(B)** distension of the perioptic subarachnoid spaces (arrowheads). **(C–F)** Sinus vein stenosis was assessed using MRI venography, here illustrated by MRI venography from four of the included patients with IIH. The image shows the transverse sinus (TS), sigmoid sinus (SS), and the superior sagittal sinus (SSS).

The MRI biomarkers of CSF disturbance retrieved from the database included the presence of empty sella (Figure 2A) and distension of the perioptic subarachnoid spaces (Figure 2B).

#### Imaging Signs of Sinus Vein Stenosis

Imaging data about signs of sinus vein stenosis retrieved from the database included MRI with venography (Figures 2C–F). This hospital has no routine for venous pressure recordings or measurements of pressure gradients over a suspected sinus vein stenosis. Therefore, MRI/MRI venography was utilized for visualization of sinus vein stenosis and signs of intracranial venous hypertension.

#### Continuous Overnight ICP Measurements

Results of overnight ICP monitoring retrieved from the database included an overnight average of pulsatile ICP (mean ICP wave amplitude; MWA), a percentage of MWA > 5 mmHg, and an average of static ICP (mean ICP), and a percentage of mean ICP > 15 mmHg.

**Figure 3:**
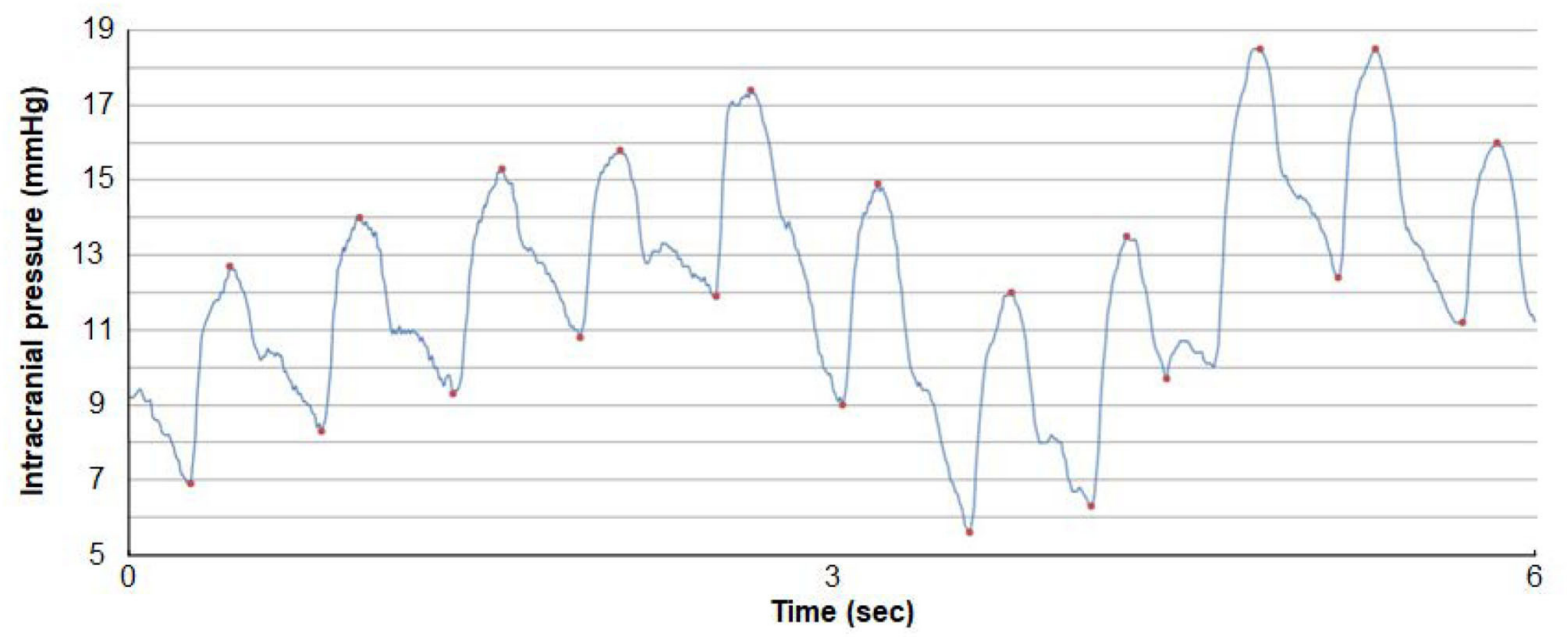
A continuous ICP signal measured from an ICP sensor placed in the frontal parenchyma of an individual with IIH. The level of ICP is shown on the y-axis and time on the x-axis. Both the pulsatile and static ICP are derived from the same ICP signal. The pulsatile ICP is described as the Mean Wave Amplitude (MWA) that refers to the pressure changes occurring during the cardiac cycle, that is, the increase in pressure from diastolic minimum pressure to systolic maximum pressure (here illustrated by the red dots). The mean ICP refers to the static ICP, that is, the absolute pressure measured against a reference pressure. The MWA and the mean ICP are determined over consecutive 6-s time windows; here mean ICP is 12.2 mmHg and MWA 6.4 mmHg. Image: Sensometrics Analytics, dPCom, Oslo, Norway.

The procedure of ICP monitoring has been described before (Eide and Kerty, 2011). In short, an ICP sensor (Codman MicroSensor^TM^, Johnson & Johnson, Raynham, MA, USA) was introduced 1–2 cm into the frontal cortex parenchyma in local anesthesia *via* a skin incision frontally on the right side, a small burr hole, and a minor opening in the dura. In the present IIH patients, overnight ICP monitoring had been performed as previously described (Eide and Kerty, 2011), utilizing a computerized software (Sensometrics software, dPCom, Oslo). The ICP signals were sampled at 200 Hz, providing an online analysis of the pulsatile ICP as mean ICP wave amplitude (MWA), and the static ICP (mean ICP) every consecutive 6-s time window (Figure 3). The software provides online trend plots and average values of the ICP scores. In general, as an indication for shunt surgery, the department's routine is the use of results of diagnostic ICP monitoring in addition to the patient history, clinical and neuro-ophthalmological findings, and imaging findings. Regarding ICP thresholds, we have defined abnormal static ICP as the average mean ICP during over-night monitoring being >15 mmHg (Eide and Kerty, 2011) or >15 mmHg in >50% of recording time, and abnormal pulsatile ICP during over-night monitoring as average MWA > 4 mmHg and/or MWA > 5 mmHg in >10% of recording time (Eide and Sorteberg, 2010; Eide and Kerty, 2011).

#### Ultrastructural Examinations of Brain Biopsy Samples

The results from histopathological analyses of brain tissue specimens from IIH and REF subjects retrieved from the database specifically addressed changes in the cell types constituting the glia-neuro-vascular interface: Glial cells, AQP4 expression at astrocytic end-feet, neurons, pericytes, basement membrane, and BBB integrity.

We described previously the surgical procedure of obtaining a brain biopsy (Eide et al., 2016). In short, a minor opening was made in the dura and a biopsy (0.9 ×10 mm) aspirated through a disposable Nashold Biopsy Needle (Integra Radionics, Burlington, MA, USA), introduced immediately below the cortical surface. This biopsy procedure provides for atraumatic and standardized biopsy sections. The brain biopsies were prepared for both light microscopy (LM) and transmission electron microscopy (TEM). The cortical biopsy was obtained at the same site where the ICP sensor or ventricular catheter was introduced.

The technical procedures concerning the handling of the brain biopsies and analysis of ultrastructure at the glia-neuro-vascular interface have previously been described in detail (Eide et al., 2016, 2021a; Eidsvaag et al., 2018; Hasan-Olive et al., 2019c).

##### Light Microscopy

The technical procedures for handling, processing, and analysis of brain tissue for LM have been described before (Eide et al., 2016; Hasan-Olive et al., 2019c). The tissue specimens comprising the superficial three cortical layers (Layers 1–3) were fixed in buffered 4% paraformaldehyde for 2 days in the cold, dehydrated, and embedded in paraffin. Tissue blocks were sectioned at a thickness of 6 μm. After deparaffinization and rehydration, the sections were immersed in aqueous hydrogen peroxide for about 10 min to quench endogenous peroxidase activity. Antigen retrieval was done by immersion in proteinase K working solution for 10 min at pH 8.0; the subsequent blocking was achieved with normal horse serum before incubation overnight with the primary antibody at 4°C. After rinsing, a biotinylated antibody was applied overnight at 4°C (Vectastain Universal Elite Kit; Vector Labs. Inc., Burlingame, CA, USA), followed by an avidin-biotin complex. Visualization was obtained with a 3,3′-diaminobezidine peroxidase substrate kit (Vector). The sections were counterstained with hematoxylin and examined by LM after mounting and being cover-glassed.

Astrocytes were identified by their expression of the anti-gliofibrillary acidic protein (GFAP) and aquaporin-4 (AQP4), activated inflammatory cells (microglia) by the antibody Cluster of Differentiation 68 (CD68), nerve cell damage by the antibody neurofilament heavy (NF-H), and extravasated fibrin(ogen) was detected by an antibody revealing both fibrinogen and fibrin (polyclonal, 1:400, Dako, A0080) as a measure of BBB dysfunction. The following primary antibodies were used: anti-gliofibrillary acidic protein (GFAP; 1:3000, mouse monoclonal clone GA5, Sigma); anti-AQP4 (polyclonal produced in rabbit against a recombinant protein tag, 1:1000, Sigma); anti-neurofilament heavy (NF-H, monoclonal against phosphorylated and non-phosphorylated neurofilaments, clone N52,1:500, Sigma); anti-CD68 (monoclonal clone KP1, 1:200;DAKO); and anti-fibrin(ogen) (polyclonal, 1:400, A0080, Dako A/S, Glostrup, Denmark). Reference sections had been processed in parallel, and the specificity of the immunohistochemical reaction was checked by the omission of the primary antibody.

**Figure 4:**
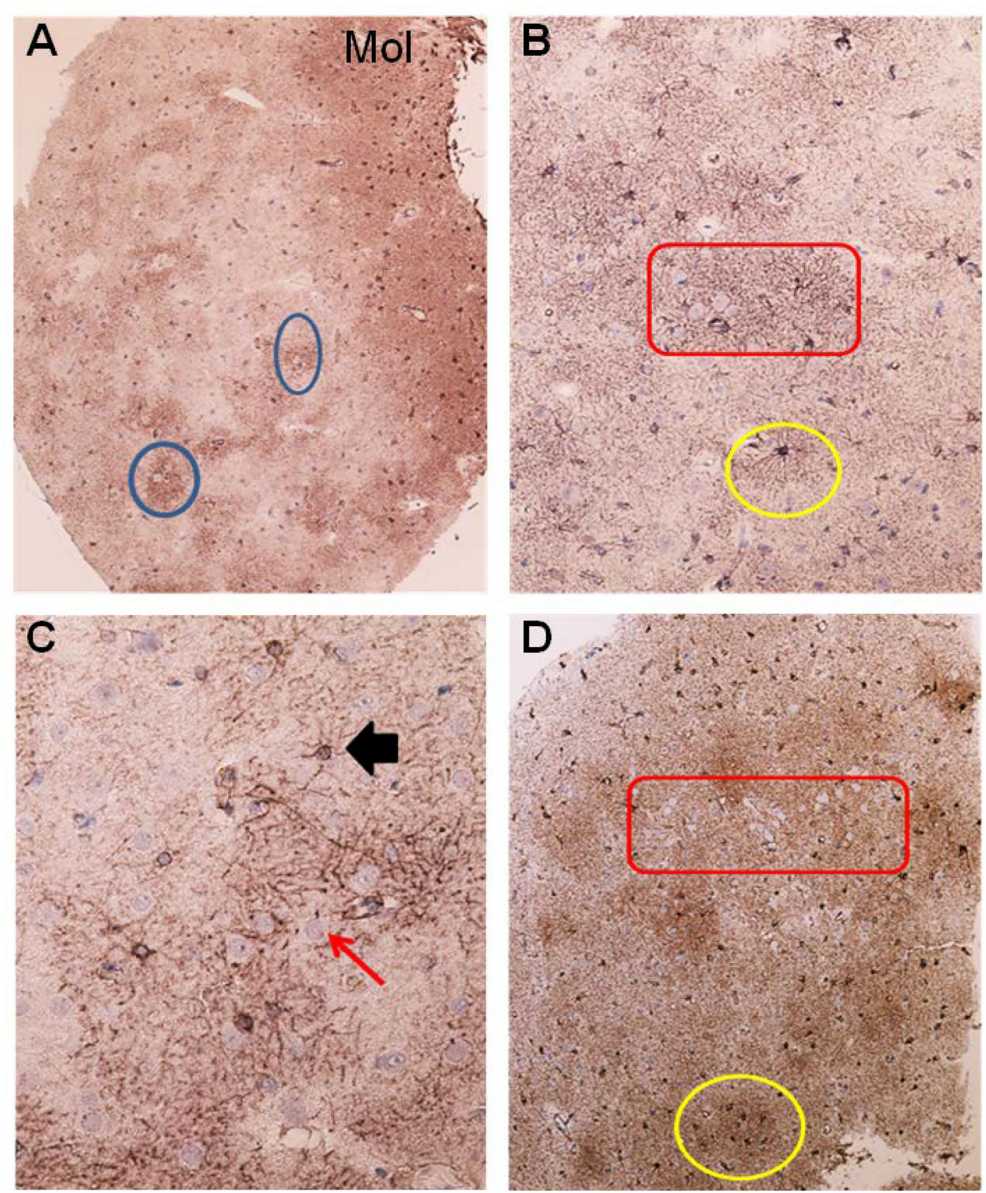
Astrocytes visualized by GFAP IR in brain cortex biopsies from IIH patients. **(A)** Low magnification of a section of brain biopsy reveals a high prevalence of astrocytes in the subpial molecular layer (Mol; layer 1, also named Chaslin's layer) looking like clusters of dark dots. Patches of astrocytes enclosing neurons are seen in cortical layers 2/3 (blue circles). **(B)** Clusters of hypertrophic astrocytes are seen enclosing unstained neurons (encircled by a red line). An apparently normal-looking astrocyte (outlined by yellow circle) with radiating slender processes without having any processes from adjacent glial cells entering its domain. **(C)** Neurons (red arrow) in the center of the specimen are enclosed by astrocytes. Note that processes from adjacent astrocytes enter their neighbors' domains as well as having increased branching (black arrow). The GFAP-IR expression is low outside the patch of neurons and astrocytes. **(D)** A low magnification micrograph of the superficial cortex layers shows a cluster of neurons forming a patch surrounded by mainly glia cells (encircled by a red line). Adjacent cluster of astrocytes enclose only few neurons (yellow circle).

**Figure 5:**
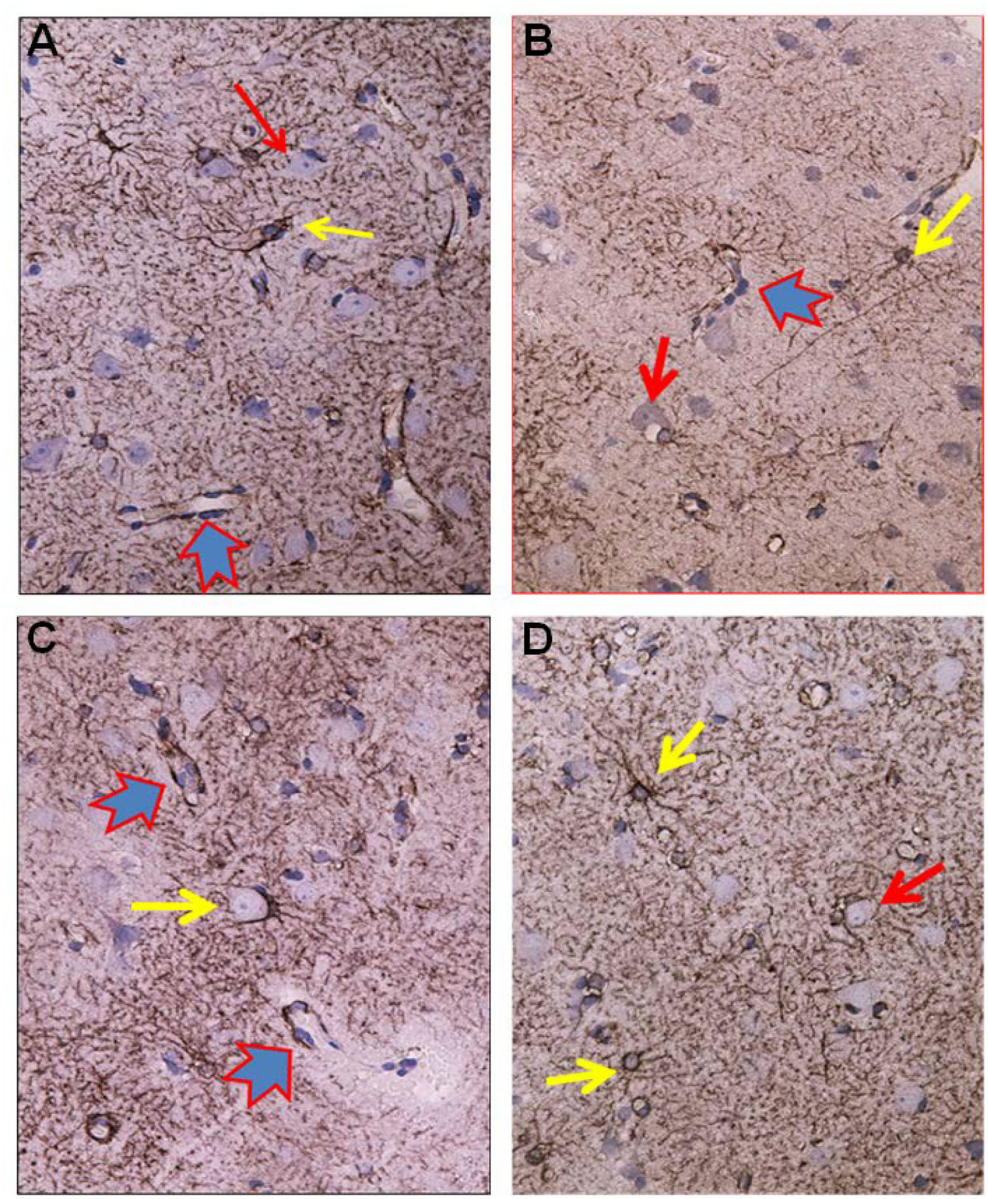
Visualization of astrocytes by GFAP-IR in cerebral cortex of IIH patients. **(A)** GFAP-IR *is* demonstrable forming an elaborated meshwork of processes and membranes. Note the swelling of the perivascular astrocytic end-feet along small blood vessels (red-blue arrow). Nerve cell marked by red arrow and astrocytes marked by yellow arrow. **(B)** Enlarged astrocyte end-feet, filled up by GFAP-IR material, were demonstrable along small blood vessel. The function, effects and importance of such enlarged GFAP-IR end-feet accumulations require further investigation. **(C)** The large nerve cell in the center (yellow arrow) is delimited by astrocyte membranes along its cell body. This is characteristic for synaptic stripping where the GFAP IR membrane continuously encloses half of the perikaryal circumference of the nerve cell body. Note that GFAP-IR profiles are demonstrable in only parts of the cortex specimen. Enlarged GFAP-IR end-feet marked by red-blue arrows. **(D)** Examples of close approximation between the astrocyte processes and the nerve cells. Further, the intensely stained GFAP-IR processes radiate from the astrocyte cell body, divide, and additionally entered neighbor cell domains. Red arrow toward nerve cell and yellow arrows toward astrocytes.

**Figure 6:**
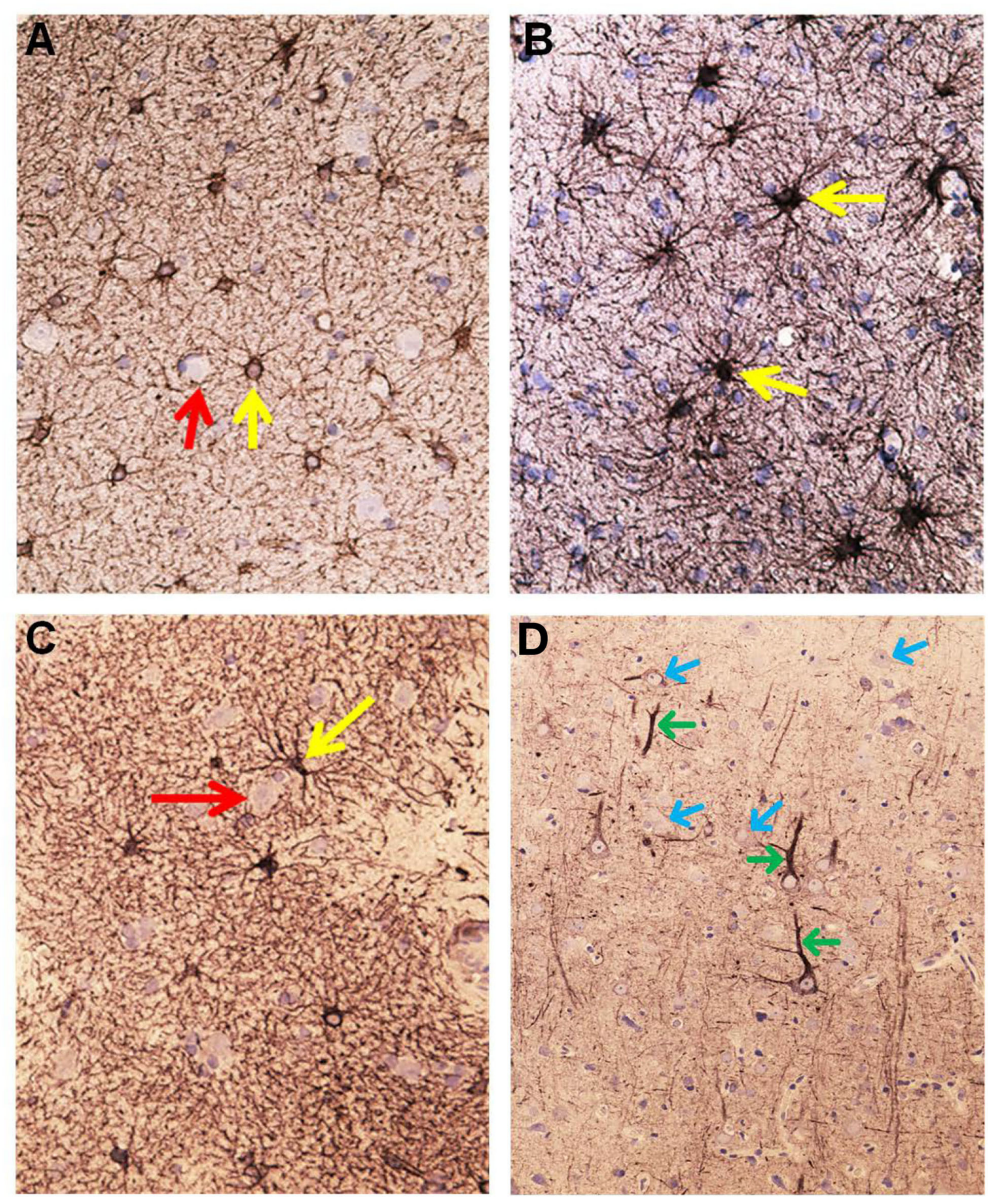
Aspects on the dynamics of astrocytes and neurons in the cerebral cortex of IIH patients. **(A)** Section of the subpial molecular cortex layer, which is dominated by astrocytes and have only scattered nerve cells. The GFAP-IR astrocytes (yellow arrow) are star-shaped with mainly slender radiating and non-dividing processes. Scattered nerve cells (red arrow) are seen in the neuropil. This illustrates the normal structure of cortex layer 1. **(B)** The astrocytes in this specimen of layer 1 are hypertrophic and their processes have abundancy of glio-filament (yellow arrow). The density of the GFAP-IR meshwork is increased in **(B)** as compared to **(A)**. **(C)** A dense network of branching and prominently GFAP-IR processes of impressive density. **(D)** In cortex layers 2/3 of an IIH subject, NF-H-IR expression occurs in damaged or dying neurons (green arrows), while living neurons remain colorless (blue arrows). This indicates neuronal damage in IIH.

**Figure 7:**
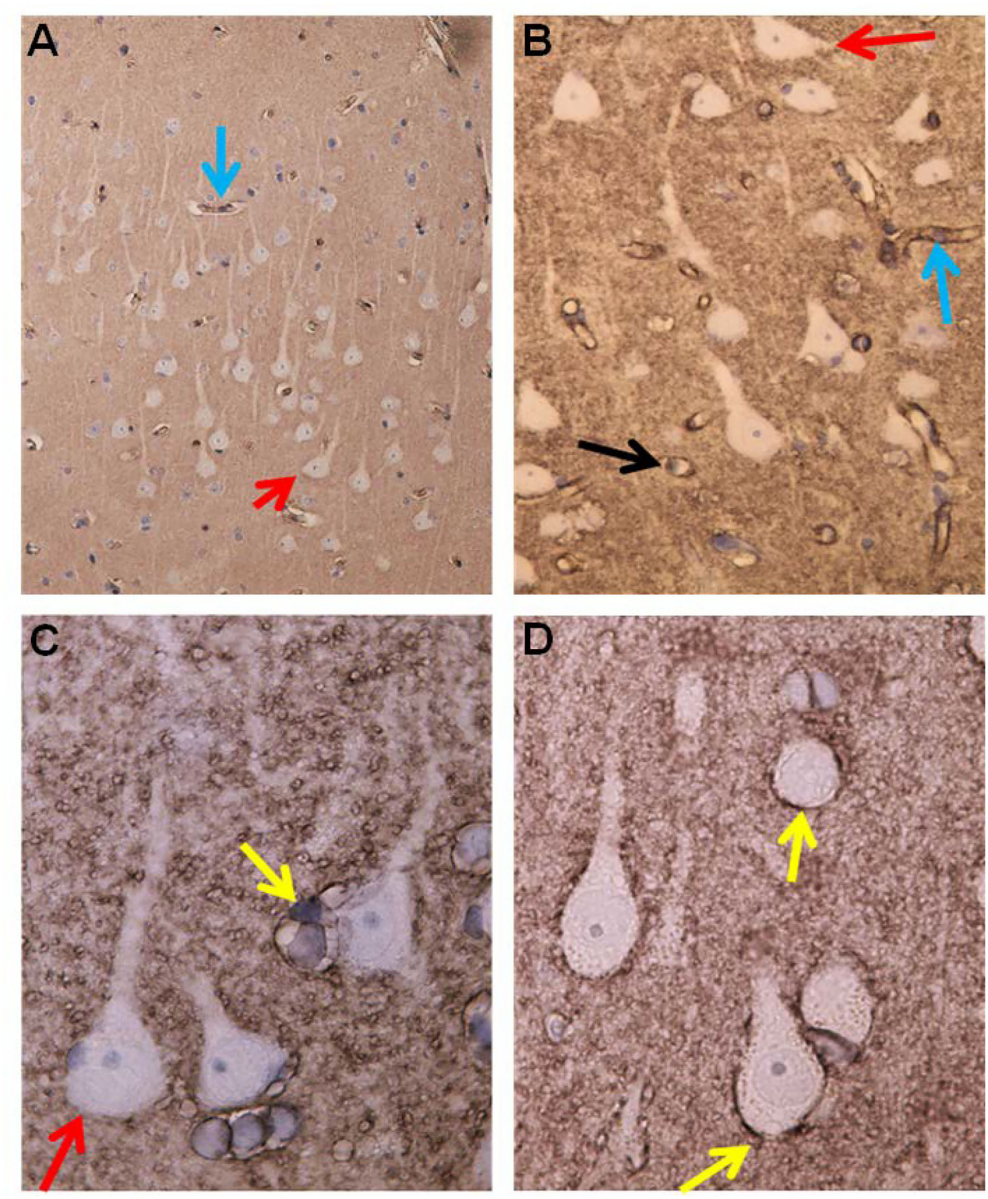
Aquaporin 4 (AQP4)—IR in cerebral cortex of IIH subjects. **(A)** In cortex layers 2/3 the neurons (red arrow) lack AQP4-IR. In contrast, the cell bodies and processes of astrocytes show moderate to strong AQP4-IR. Small blood vessels are distinctly outlined due to high AQP4 IR in the astrocyte end-feet (blue arrow). **(B)** This Figure illustrates that the small cerebral vessels are distinctly outlined due to the prominent AQP4-IR in astrocyte end-feet. Note that the perivascular end feet lining blood vessels are more intensely reactive than the neuropil. **(C)** Pyramidal nerve cells (red arrow) could be observed outlined by seemingly continuous AQP4-IR along the neurilemma, as noticed for many neurons (reference patient). In contrast, no enclosing continuous rim of AQP4-IR was observed in IIH. **(D)** The significance of the different patterns of AQP4 IR in end-feet in different locations ought to be further elucidated.

**Figure 8:**
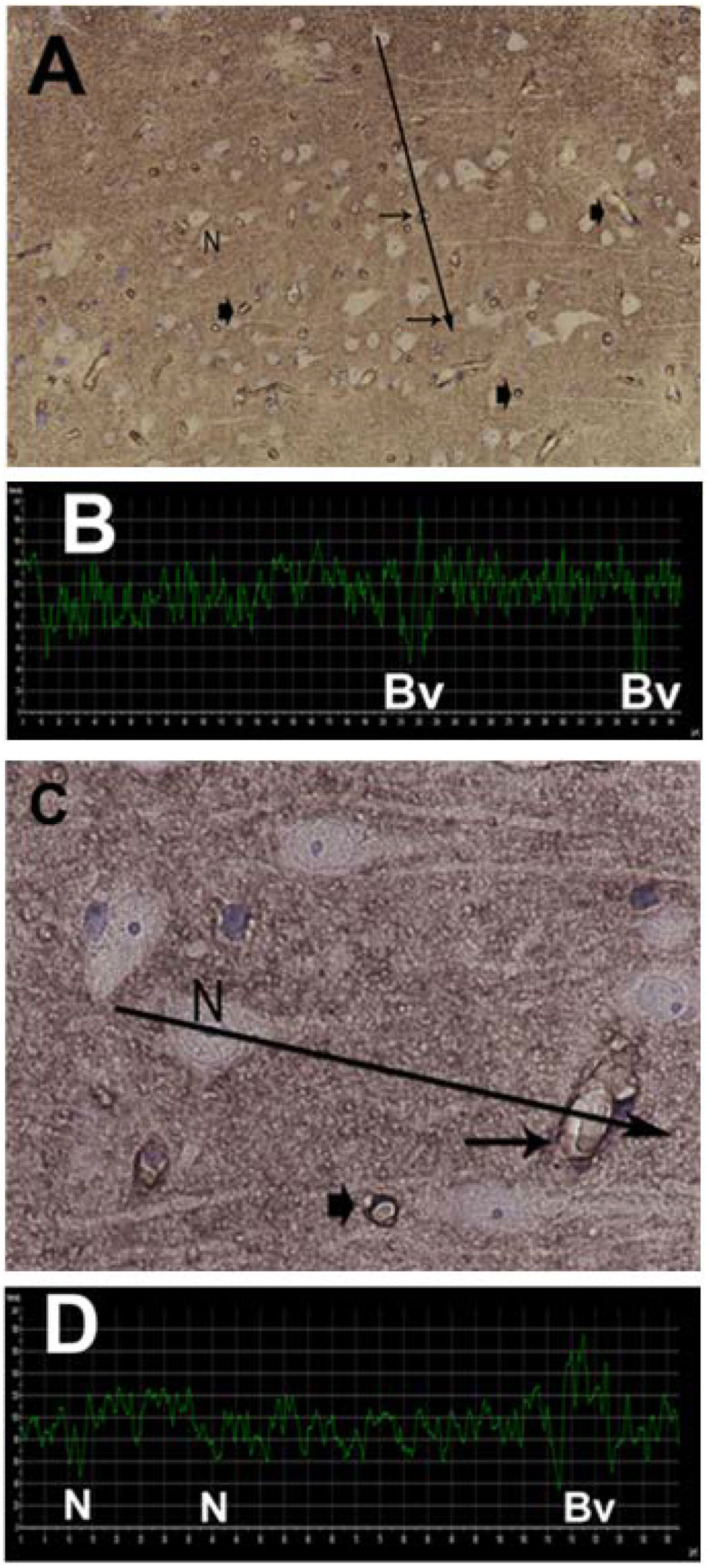
Semi quantitative densitometry determination of AQP4 IR in cerebral biopsies in IIH patients. **(A)** The pattern of AQP4 IR is revealed. The densitometric measurement in **(A)** along a line is shown in **(B)**. Low transmission of light discloses strong AQP4 IR. The nerve cells are non-immunoreactive contrasting to the prominent AQP4-IR in the neuropil. Densitometric analysis enables mapping of the sites with reactive and non-reactive cell structures. **(C)** The relative intensity of the AQP4 IR can be mapped at the cellular level enabling more detailed analysis. The neurons are non-reactive. The perivascular astrocytic end-feet show prominent AQP4 IR. **(D)** The densitometric measurement.

Glial cells were examined for the presence of astrogliosis, astrocyte hypertrophy, and loss of astrocyte domains (Figures 4–6). Furthermore, the expression of AQP4 perivascular, as well as in neuropil, was determined (Figure 7). Semi-quantification of AQP4 expression was done using densitometry analysis that provides an estimate of transmitted light, expressed as anarbitrary units (Figure 8). Lower arbitrary units imply increased AQP4 expression. Nerve cell bodies could be disclosed to be extensively delimited by astrocyte membranes with high AQP4IR (Figure 7).

Morphometry was done to determine the percentage area of either GFAP or CD68 immunoreactivity (3 areas per biopsy specimen, 273 × 410 μm), utilizing a Nikon Eclipse Ni microscope, a DS-Ri2 camera, and NIS element B.V4.3 program (Nikon, Tokyo, Japan), and a Leica QWin Pro system (Leica Biosystems GmbH, Wetzlar, Germany).

The semi-quantification and morphometry procedures were performed by one person (HAH) who was blinded to the diagnosis of the patients.

Neuronal changes were assessed by the expression of neurofilament-H (NF-H) and by the identification of synaptic stripping. The NF-H IR was indicative of axonal damage characteristic for damaged neurons, while the lack of NF-H IR was typical for normal neurons (Figure 6D). Synaptic stripping was characterized by nerve cell bodies being delimited by a GFAP IR membrane enclosing at least half of the nerve cell body sectioned through the nucleus (Figure 5C).

**Figure 9:**
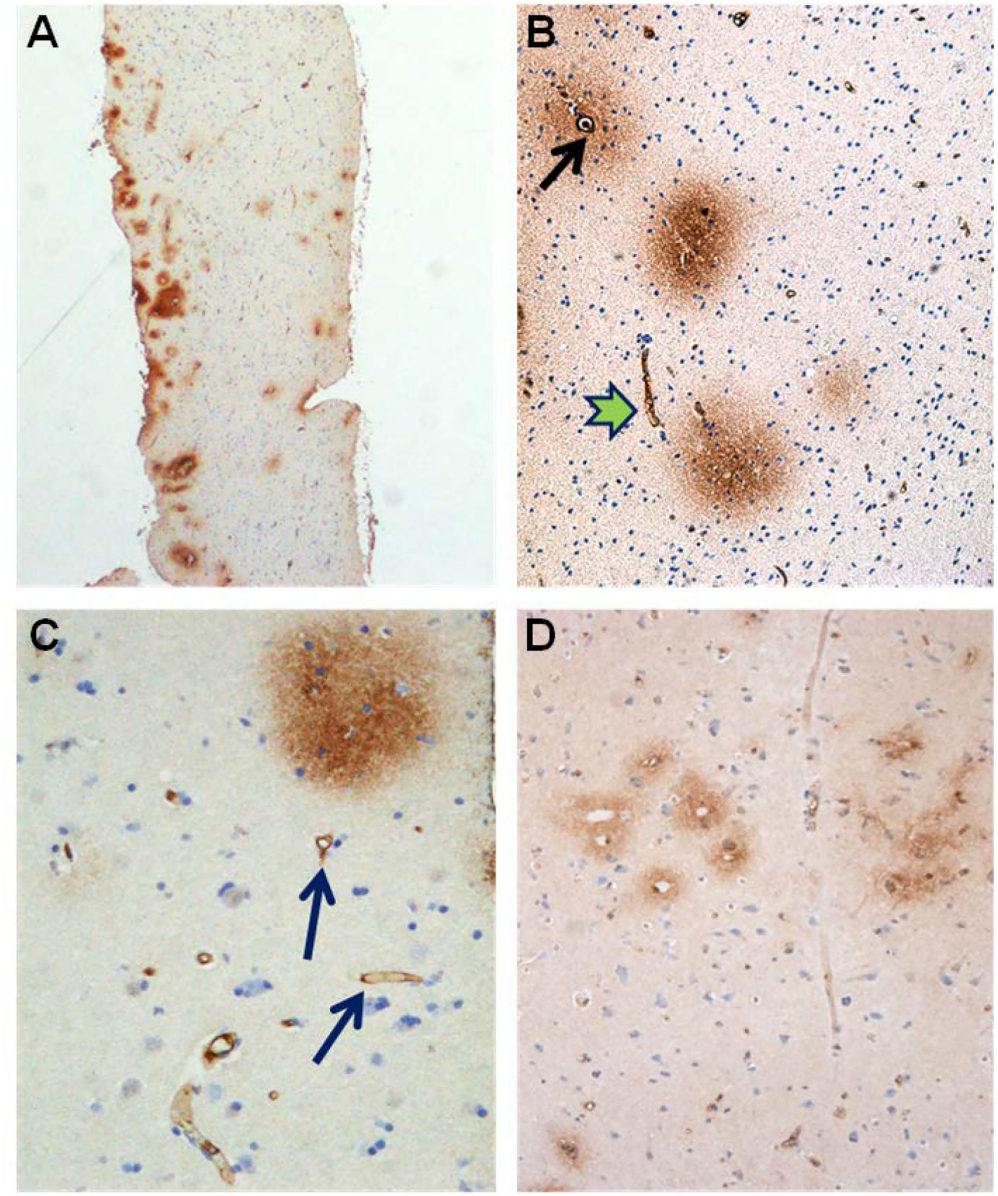
Evidence of BBB dysfunction in IIH subjects. **(A)** The cerebral cortex biopsies comprised cortex tissue that was dissected and fixed by immersion within minutes to achieve best possible preservation. Extravasations of blood products were revealed by immunohistochemical demonstration of both fibrin and fibrinogen, i.e., the blood proteins detected by the used antibody. **(B)** Leakage from a small blood vessel (black arrow) in the center of extravasated fibrin(ogen). Note the vessel wall at the top is retaining blood proteins. **(C)** Fibrin (ogen) is demonstrable both extravasated (upper right corner) and in the walls of small brain vessels (arrow). **(D)** A cluster of small blood vessels is surrounded by leaked fibrin (ogen).

Alterations in capillaries were examined by identifying degenerating pericytes processes and degeneration of the basement membrane visualized as vacuolization and degenerative splitting. A cortical capillary was defined as a vascular structure with an inner diameter <8 μm with the lumen bordered by a thin layer of endothelial cells (1–2 per circumference), delimited abluminally by a basement membrane (BM), and enclosed by pericytes (cell body and/or processes) (Mathiisen et al., 2010; Alberts et al., 2014; Winkler et al., 2014; Sweeney et al., 2016). Signs of degeneration included degenerating pericytes, and increased occurrence of vacuoles was graded as follows: None = 0, slight = 1–2, moderate 3–4; severe ≥ 5, and/or vacuoles enclosed more than half of the circumference of the capillaries. The astrocytic perivascular processes were identified by their position along the abluminal border of the basement membrane, enclosing the microvessels. Leakage of the BBB was examined by determining the area of fibrin(ogen) immunoreactivity in the neuropil (Figure 9).

##### Transmission Electron Microscopy

We have described the routine for TEM in IIH (Eidsvaag et al., 2018; Eide et al., 2021a). The tissue specimens comprising at least the three deeper layers of the cerebral cortex (Layers 4-6) were immersion fixed in 0.1-M phosphate buffer containing 4% paraformaldehyde and 0.25% glutaraldehyde and kept in a fridge (4°C) overnight. Then, they were transferred to the same fixative diluted 1/10 in phosphate buffer and stored in the solution until further processing. Small blocks from the biopsies were cut, undergoing freeze substitution and infiltration in a Lowicryl HM20 resin (Polysciences Inc., Warrington, PA, USA, Cat15924). The sections were counterstained with uranyl acetate during the cryosubstitution steps before the Lowicryl embedding. Using a Reichert ultramicrotome (Wien, Austria), sections of 80 nm were cut, and mounted on nickel grids. An FEI Tecnai^TM^ 12 transmission electron microscope (FEI Company, Hillsboro, OR, USA) was applied for TEM recording; images were acquired with the analySIS image analysis software (Soft Imaging Systems GmbH, Münster, Germany).

**Figure 10:**
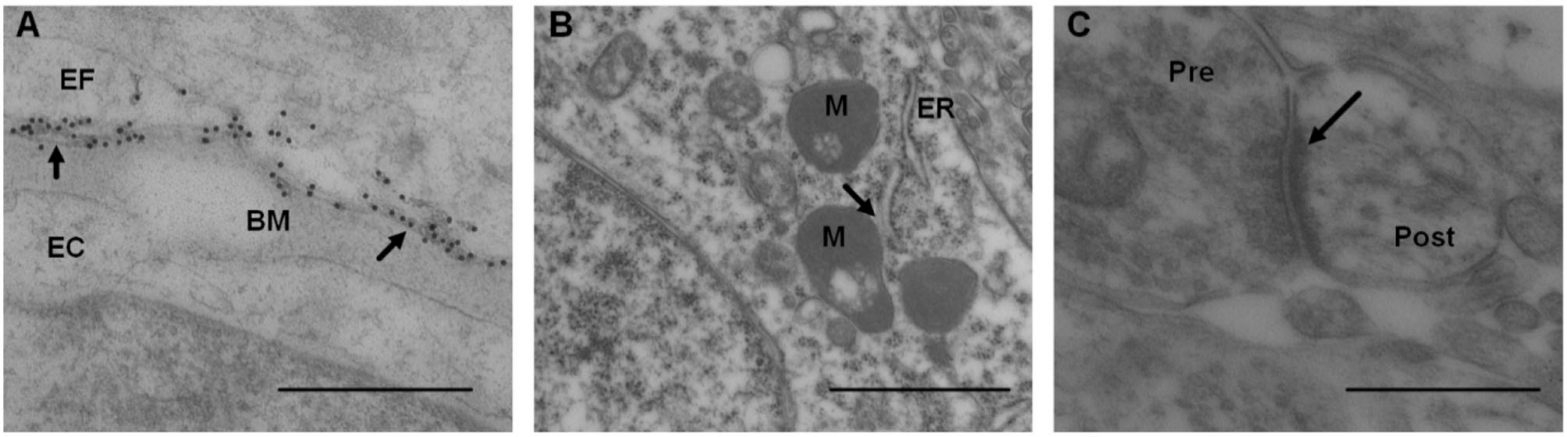
Transmission electron microscopy images of biopsy from right frontal cortex of IIH patients. **(A)** Expression of AQP4 was shown by immunogold labeling of AQP4 in perivascular astrocytic end-feet (EF) facing basement membrane (BM) and endothelial cells (EC); arrows indicate contact toward BM. Scale bar 500 nm. **(B)** Distance between mitochondria (M) and endoplasmic reticulum (ER) in patients with IIH, denoted mitochondria-ER-contact sites (MERCs; arrow). Shortened distance is indicative of pathology. Scale bar 1 μm **(C)** Post-synaptic density (PSD) length (arrow) in IIH, with pre (Pre) and post-synaptic (Post) nerve terminals. Reduced PSD length is indicative of pathology. Scale bar 500 nm.

The AQP4 expression was analyzed as the linear density of AQP4 toward endothelium (Figure 10A), as previously described (Hasan-Olive et al., 2019c). Mitochondrial-ER contact sites (MERCs) were analyzed as the length of the ER toward the mitochondrion (Figure 10B), as previously described (Eide et al., 2021a). The shortest distances between ER and mitochondria were measured at equal distances along with this contact site. For each individual, the length of mitochondria/ER contact was measured, and the average distance between mitochondria and ER was calculated for the particular length. The average of the measurements of the distance between mitochondria to ER was referred to as MERCs distance. Shortened distance between mitochondria and ER is indicative of pathology (Leal et al., 2018; Stacchiotti et al., 2018). Postsynaptic densities (PSDs) were identified in dendritic spines and measured by their length of electron-dense appearance (Figure 10C), as previously described (Eide et al., 2021a). The postsynaptic density is a measure of synaptic strength.

### Statistical Analysis

The statistical analyses were done with the SPSS software version 27 (IBM Corporation, Armonk, NY). Differences between IIH and REF cohorts were determined using independent sample *t*-tests for continuous data, and by Pearson's chi-square test for categorical data. The statistical significance was accepted at the 0.05 level.

## Results

### Occurrence of Indices in Patients With IIH About the Underlying Pathophysiology

The study included 13 consecutive patients with IIH (11 female and two male; average age 33.1 ± 10.7 years) who fulfilled the inclusion criteria. An overview of variables retrieved from the database is provided in Table 1. The included patients with IIH fulfilled the diagnostic criteria of IIH (Friedman et al., 2013; Mollan et al., 2018): (A) Papilledema. (B) Normal neurological examination (except for 6th cranial nerve affection). (C) Normal magnetic resonance imaging (MRI), i.e., absence of hydrocephalus, mass, structural lesions, or meningeal enhancement, with the exclusion of venous thrombosis. (D) Normal CSF composition. (E) Elevated lumbar opening pressure (>25 mmH_2_O). Notably, subjective cognitive impairment was reported by 6/13 (46%) of patients with IIH.

**Table 1:** Variables indicative of idiopathic intracranial hypertension (IIH) pathophysiology in the 13 patients with definite IIH.

	**IIH patient**												
	**1**	**2**	**3**	**4**	**5**	**6**	**7**	**8**	**9**	**10**	**11**	**12**	**13**
**Demographic**													
Gender	F	F	F	F	F	F	F	F	F	M	F	F	M
Age (yrs)	38	30	22	34	24	48	37	47	22	21	24	52	31
BMI (kg/m^2^)	29	33	29	34	28	23	26	33	38	35	37	39	23
**Symptoms**													
Headache	1	1	1	1	1	1	1	1	1	1	1	1	1
Papilledema/Visual deficits	1	1	1	1	1	1	1	1	1	1	1	1	1
Tinnitus	–	–	1	1	1	–	–	–	–	–	–	1	1
Cognitive impairment	1	1	–	–	1	1	1	–	–	–	–	1	–
**MRI-evidence of CSF disturbance**													
Empty sella/distended													
Perioptic subarachnoid space	1	–	–	–	1	1	–	–	1	–	–	1	1
**MRI-Evidence of venous obstruction**													
Sinus vein stenosis	–	–	–	–	–	–	–	–	–	–	–	–	–
**Overnight ICP**													
Abnormal MWA	1	1	1	1	1	1	1	1	1	1	1	1	1
Abnormal mean ICP	1	–	–	–	–	1	–	–	–	–	–	–	1
**Ultrastructural abnormality at the glia-neuro-vascular interface**													
Glial cells													
Astrocyte hypertrophy/loss of astrocyte domains	1	1	1	–	1	1	–	1	1	1	1	1	1
Patchy astrogliosis	1	1	–	–	–	1	–	–	1	–	1	1	–
Neurons													
Neurofilament-H	1	1	–	–	–	1	–	–	–	–	1	–	–
Synaptic stripping	1	–	–	–	–	1	–	1	1	–	1	–	–
Capillaries													
Degeneration of pericyte processes	1	–	–	–	1	1	1	1	1	1	1	–	–
BM vesicles/splitting	1	–	–	–	1	1	1	1	1	1	1	–	–
BBB leakage	1	–	–	–	1	1	1	1	1	1	1	1	1

*BBB, blood-brain-barrier; BM, Basement membrane; BMI, body mass index; ICP, Intracranial pressure; MWA, mean; ICP, wave amplitude*.

The IIH cohort had a disease history of 5.1 ± 3.4 years. Before surgical workup, they had undergone conservative measures, including weight reduction, and medical treatment (acetazolamide in 10/13 patients, topiramate in 2/13 of patients, and furosemide in 2/13 patients).

Magnetic resonance imaging (MRI) evidence of CSF disturbance indicated by empty sella and/or distended perioptic subarachnoid spaces were observed in 6/13 (46%) of patients with IIH (Table 1). Most importantly, in this series of 13 patients with definite IIH, none presented with MRI signs of sinus vein stenosis (Table 1).

**Figure 11:**
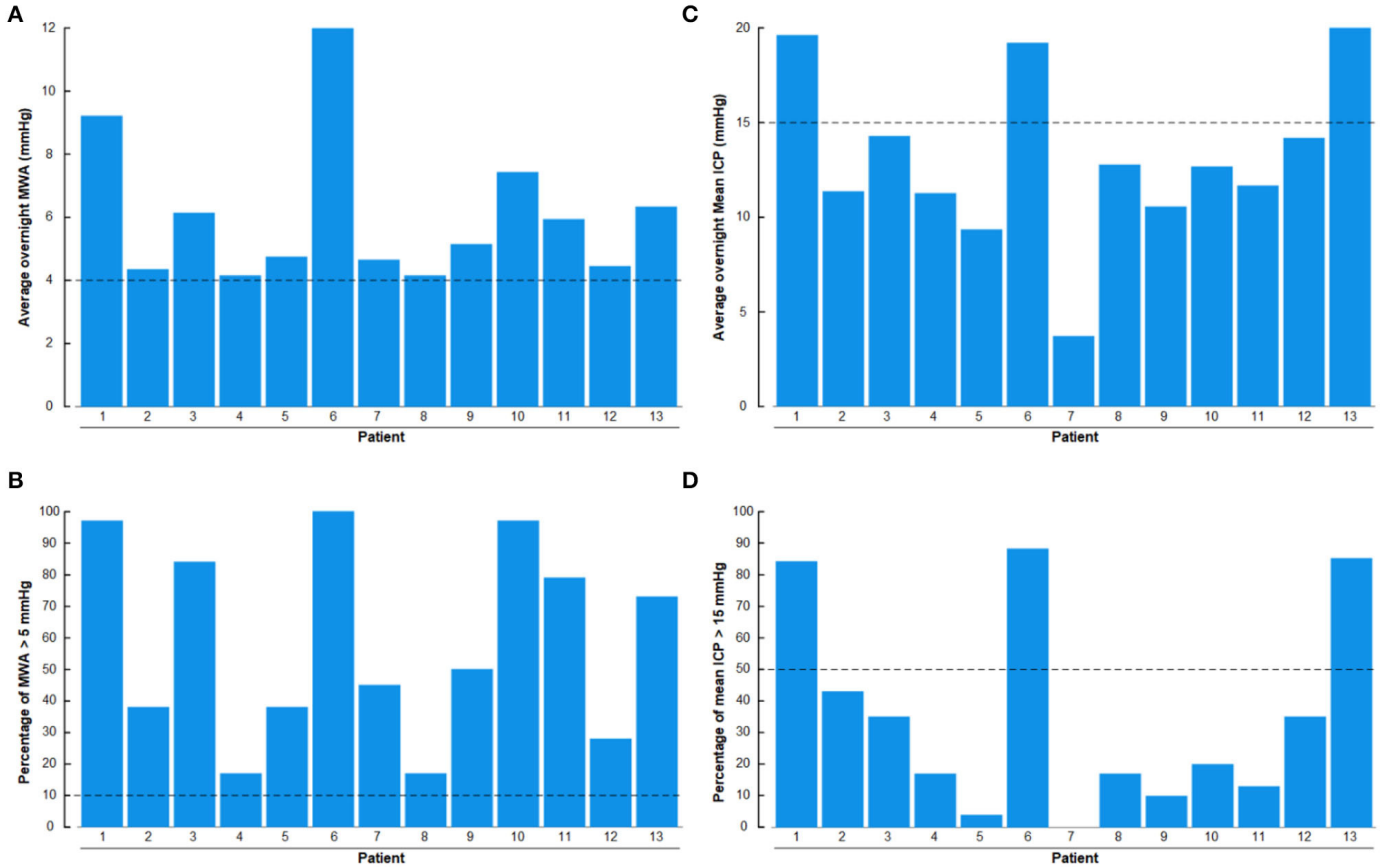
The overnight recordings of MWA and mean ICP in the 13 Patients with IIH included in the study are shown on an individual basis. **(A)** Overnight average of MWA. **(B)** Overnight percentage of MWA > 5 mmHg. **(C)** Overnight average of mean ICP. **(D)** Overnight percentage of mean ICP > 15 mmHg. The horizontal lines indicate upper normal thresholds as defined by the first author: **(A)** Overnight MWA > 4 mmHg. **(B)** Overnight 10% of MWA > 5 mmHg. **(C)** Overnight Mean ICP > 15 mmHg. **(D)** Overnight 50% of Mean ICP > 15 mmHg.

There were abnormal pulsatile ICP in 13/13 (100%) IIH subjects, shown as overnight MWA scores of >4 mmHg (Figure 11A) or overnight percentage of MWA > 5 mmHg in > 10% of recording time (Figure 11B). Abnormal mean ICP, either defined as overnight mean ICP >15 mmHg (Figure 11C) or overnight mean ICP > 15 mmHg in >50% of recording time (Figure 11D), was observed in 3/13 (23%) of IIH subjects.

The brain biopsies were not accompanied by adverse consequences. Abnormal ultrastructure at the glia-neurovascular interface was demonstrated in 12/13 (92%) of patients with IIH; only case #4 presented with no apparent abnormal ultrastructure. Table 1 provides an overview of some ultrastructural findings in the patients with definite IIH. Continuous variables cannot be dichotomized as abnormal/normal and were not included in Table 1. The brain biopsies were obtained from right frontal gray matter in the 13 patients with IIH. The 12 REF subjects had undergone surgery for epilepsy (*n* = 9), clipping of a cerebral aneurysm (*n* = 2), or resection of a malignant brain tumor (*n* = 1). The reference tissue specimens were obtained from the gray matter of the frontal cortex in three, temporal cortex in seven, parietal cortex in one, and occipital cortex in one subject.

### Comparisons of Semi-quantitative Ultrastructural Variables

The semi-quantitative variables of ultrastructural change at the glia-neuro-vascular interface retrieved from the database were compared between 13 Patients with IIH and 12 REF subjects, who were comparable in age and gender (Table 2). The IIH and REF cohorts differed significantly in BMI and the occurrence of the symptoms, such as headache, papilledema/visual impairment, and tinnitus (Table 2). Furthermore, the female/male distribution was 11/2 among IIH and 7/5 among REF subjects, even though the difference was non-significant.

**Table 2:** Material of patients with definite IIH and REF subjects.

	**IIH**	**REF**	**Significance**
Number	13	12	
Gender (F/M)	11/2	7/5	ns
Age mean at inclusion (yrs.)	33.1 ± 10.7	37.9 ± 11.7	ns
BMI (kg/m^2^)	31.2 ± 5.4	25.8 ± 4.3	*P* = 0.012
**Co-Morbidity**			
Arterial hypertension, *n* (%)	1 (7.7%)	2 (16.7%)	ns
Diabetes mellitus, *n* (%)	1 (7.7%)	0	ns
**Pre-Operative symptoms**			
Headache	13 (100%)	2 (17%)	*P* <0.001
Papilledema/visual impairment	13 (100%)	0	*P* <0.001
Tinnitus	5 (39%)	0	*P* = 0.016
Cognitive impairment	6 (46%)	4 (33%)	ns

*BMI, Body mass index; F, Female; M, Male. Continuous data are presented as mean ± standard deviation, and categorical data as numbers (with percentages in parenthesis). Significant differences between continuous variables were determined by independent samples t-test, and differences between categorical data were determined by Pearson's chi square test. Ns, non-significant*.

A comparison of morphology between IIH and REF subjects showed a significantly increased area of GFAP immunoreactivity, higher occurrence of patchy astrogliosis, and loss of astrocyte domains in IIH subjects (Table 3).

**Table 3:** Comparisons of structural differences at glia-neuro-vascular interface of patients with definite IIH and reference (REF) subjects.

	**IIH**	**REF**	**Significance**
Glial cells			
**Astrocytes**			
Area of GFAP immunoreactivity (%)	13.2 ± 3.1	7.3 ± 2.7	*P* < 0.001
Patchy astrogliosis (yes/no)	6/7	1/10	*P* = 0.047
Distinct astrocyte domains (yes/no)	3/10	9/2	*P* = 0.004
**Perivascular astrocytic end-feet**			
**AQP4 at membranes facing endothelial cells**			
LM–Arbitrary units	48.7 ± 15.4	68.5 ± 7.4	*P* = 0.001
TEM–Linear gold particle density (particles/μm)	18.3 ± 3.1	17.9 ± 4.2	ns
**AQP4 at membranes facing neuropil**			
LM–Arbitrary units	116.5 ± 3.9	115.3 ± 7.7	ns
TEM–Linear gold particle density (particles/μm)	8.2 ± 3.6	8.1 ± 2.5	ns
**Microglia**			
Area of CD68 immunoreactivity (%)	0.45 ± 0.23	0.70 ± 0.45	ns
EM markers of abnormal neuronal function			
**Neuronal soma**			
MERCs distance (μm)	0.05 ± 0.001	0.24 ± 0.04	*P* = 0.008
**Post-Synaptic density**			
Post-Synaptic density length (μm)	0.35 ± 0.05	0.63 ± 0.09	*P* = 0.001
Microvascular alterations			
**Pericyte process degeneration**			
Category (normal/focal/extensive)	0/8/0	4/4/0	*P* = 0.021
**Basement membrane abnormality**			
Vacuolization (none/slight/moderate/severe)	0/6/2/0	4/1/2/1	*P* = 0.036
Degeneration splitting (Y/N)	8/0	4/4	*P* = 0.021
**Blood-brain-Barrier leakage**			
Area of fibrin(ogen) immunoreactivity	11.2 ± 7.9	0.8 ± 1.1	*P* < 0.001

*AQP4, Aquaporin-4; CD68, Cluster of differentiation 68; EM, Electron microscopy; GFAP, Glial fibrillary acidic protein; LM, Light microscopy; MERC distance, Mitochondria-endoplasmic reticulum distance. Continuous data presented as mean ± standard deviation, and categorical data as numbers. Lower arbitrary units are accompanied with higher AQP4 expression. Significant differences between groups were determined by independent samples t-test for continuous data and by Pearson's Chi-Square test for categorical data (ns, non-significant)*.

As evaluated by LM, AQP4 immunoreactivity at perivascular borders was increased as visualized by LM, but this was not verified by TEM-utilizing immunogold labeling of AQP4 (Table 3). These observations confirm that the perivascular expression of AQP4 is not reduced in IIH subjects. regarding the possible role of neuroinflammation and microglia in IIH, no difference in CD68 expression was seen in IIH subjects (Table 3).

As indicated in Table 3, impaired neuronal function in IIH was indicated by reduced MERCs distance, as well as reduced post-synaptic density length.

Furthermore, the semi-quantitative comparisons indicated that patients with IIH present with alterations at the capillary level. The IIH subjects were characterized by more frequent degeneration of pericytes processes, as well as signs of vacuolization and degeneration of the basement membrane (Table 3). Moreover, there was marked evidence of blood-brain-barrier leakage in IIH, shown as an increased area of fibrin(ogen) immunoreactivity (Table 3).

## Discussion

The major observation of this study was that the present 13 patients with definite IIH demonstrated MRI signs of CSF disturbance in 6/13 (46%) of patients, signs of sinus vein stenosis in 0/13 (0%), abnormal pulsatile ICP in 13/13 (100%) of patients, abnormal static ICP in 3/13 (23%) of patients with IIH, and ultrastructural changes indicative of pathology at the glia-neuro-vascular interface in 12/13 (92%) of patients with IIH. Furthermore, comparisons of semi-quantitative variables of ultrastructural changes at the glia-neuro-vascular interface demonstrated abnormalities for variables related to glial cells, neurons pericytes, and capillaries in patients with IIH.

### Patients With IIH

The present cohort of 13 patients with IIH may be considered *Definite* patients with IIH. Before shunt surgery, they fulfilled the diagnostic criteria of IIH (Friedman et al., 2013; Mollan et al., 2018), and responded with clinical improvement (visual improvement and at least partial improvement of headache) following shunt surgery. It should be noted that cognitive impairment is frequently reported in IIH (Kharkar et al., 2011; Yri et al., 2014), and was reported by 6/13 (46%) of patients with IIH in this study. There is evolving evidence for olfactory dysfunction in IIH (Schmidt et al., 2012; Kunte et al., 2013; Bershad et al., 2014), but this cranial nerve dysfunction was not recorded in our registry. The present cohort included non-selected and consecutive patients with IIH, who are representative of patients with IIH referred to neurosurgery. These patients with IIH may, however, not be representative of a general cohort of patients with IIH as these patients are primarily treated conservatively before referral to neurosurgery. This IIH cohort had been treated conservatively 5.1 ± 3.4 years before neurosurgical treatment.

### CSF Disturbance in IIH

In this IIH cohort, MRI evidence for CSF disturbance visualized as empty sella or distended perioptic subarachnoid spaces was observed in 6/13 (46%) of patients. Traditionally, CSF circulation failure has been considered the main mechanism behind IIH, related to excessive CSF production and/or reduced CSF efflux. This is as well the main rationale behind the medical treatment of reducing CSF production. Hence, the medications acetazolamide and topiramate reduce CSF production by inhibiting carbonic anhydrase. It should be noted, however, that even though acetazolamide is the most widely used medication for IIH, a recent Cochrane review concluded that there is insufficient evidence to recommend or reject the efficacy of this medication (Piper et al., 2015). Among the present IIH subjects, at the time of ICP/biopsy, 10/13 (77%) used acetazolamide and 2/13 (15%) topiramate, but with lasting symptoms, and abnormal pulsatile ICP in all patients (Figure 11B; Tables 1, 2).

On the other hand, the strongest evidence in favor of CSF disturbance in IIH is the clinical response to CSF diversion surgery, either ventriculoperitoneal or lumbo-peritoneal shunting. All the present patients with IIH had normalization of visual impairment following shunt surgery and at least partial improvement of headache. Hence, reducing CSF pressure by shunting effectively improves visual function, even though lasting complaints with headaches are frequent, and the shunt revision rate in IIH is high (McGirt et al., 2004; Eide and Kerty, 2011; Eide et al., 2016). Therefore, given that normalization of CSF pressure may not be accompanied by improved headache (Eide, 2021), other mechanisms than CSF pressure, *per se*, seems to be at play. Today, there is a tendency toward treating headaches associated with IIH as migraine and recommend anti-migraine medications.

### Intracranial Venous Hypertension and Obesity in IIH

The diagnostic criteria of IIH require imaging studies demonstrating normal brain parenchyma with no hydrocephalus, mass lesions, structural lesions, meningeal enhancement, or evidence of venous thrombosis (Friedman et al., 2013; Mollan et al., 2018). These MRI findings were confirmed in the present patients with IIH.

An important hypothesis regarding IIH pathophysiology is intracranial venous hypertension caused by sinus vein stenosis (Mollan et al., 2018). It even has been proposed that the notation “idiopathic” should be omitted, based on the reasoning that intracranial venous hypertension is the main cause of the disease (Fargen, 2020). It is, however, complicating that the diagnostic criteria for the presence of significant sinus vein stenosis vary. Based on MRI venography, none of the present patients with IIH showed evidence of sinus vein stenosis and accompanying intracranial venous obstruction. Moreover, static ICP was neither markedly increased; merely 3/13 (23%) of patients with IIH presented with overnight mean ICP > 15 mmHg or overnight percentage of mean ICP > 15 mmHg in >50% of observations. Significant venous obstruction caused by sinus vein stenosis would be expected to increase the static ICP. Therefore, the present data do not add support the assumption that sinus vein stenosis is a significant player in IIH pathophysiology.

Intracranial venous hypertension may, however, be increased for other reasons in IIH. The incidence of IIH in the general adult population was 1–2 in 100,000 whereas, in adult obese fertile women, the incidence was 12–32 in 100,000 people (Kesler et al., 2014). Based on an assumption that being overweight leads to increased abdominal pressure and, thereby, increased venous pressure that subsequently increases ICP, weight reduction is the first-line treatment. In line with this, a low-energy diet in women with IIH was accompanied by less severe symptoms, reduced papilledema, and lowered ICP (Sinclair et al., 2010). In the present IIH cohort, BMI was significantly increased, and a BMI of >30 kg/m^2^ was found in 7/13 (54%) of patients.

### ICP Measurements in IIH

The present observations in shunt-responsive IIH subjects of abnormal pulsatile ICP in 13/13 (100%) of subjects, but abnormal static ICP in 3/13 (23%) patients compared with previous observations in patients with IIH refractory to conservative-medical treatment, namely abnormal pulsatile ICP despite normalized static ICP (Eide and Kerty, 2011; Eide, 2021). For years, we have recorded ICP in IIH and have measured ICP *via* a sensor placed in the brain parenchyma since parenchymal ICP measurements may be considered the gold standard. Using dedicated software, we have determined the overnight pulsatile ICP as the mean ICP wave amplitude (MWA), in addition to the static ICP (mean ICP). From this experience, we have established upper normal thresholds for MWA (Eide and Kerty, 2011; Eide, 2021). Since few studies have reported the results of continuous over-night ICP monitoring in IIH, we have adopted upper normal thresholds of mean ICP from the experience of others; usually a mean ICP of <15 mmHg has been considered normal (Corbett and Mehta, 1983; Whiteley et al., 2006; Bono and Quattrone, 2007), but ICP has been estimated from measurements of lumbar CSF opening pressure with the patient in the lateral decubitus position. The upper normal pressure values ranging between 200 and 250 mm H_2_O (14.7–18.4 mm Hg) (Corbett and Mehta, 1983). UK physicians treating IIH consider a gray zone between 250 and 300-mm CSF to be disease defining (Wakerley et al., 2020). Even though the diagnosis of IIH relies on lumbar CSF pressure (Mollan et al., 2018), it is important to bear in mind that there are definite limitations to estimating ICP from lumbar puncture (Eftekhari et al., 2019). The lumbar CSF pressure depends on many factors; for example, the position of the lumbar region relative to the head causes hydrostatic pressure differences to affect the lumbar CSF pressure. In addition, breath-holding hyperventilation or Valsalva related to pain, discomfort, and anxiety all affect the lumbar CSF pressure. Furthermore, since the lumbar CSF pressure changes over time, it has been suggested to measure lumbar CSF pressure over longer periods (Torbey et al., 2004). Therefore, the ICP and lumbar CSF pressure are not identical, even with the patient in the horizontal position and absence of CSF blocks. In line with this, simultaneous measurements of the CP from a parenchymal sensor and from the lumbar CSF compartment demonstrated different pulsatile and static ICP scores (Eide and Brean, 2006). Accordingly, the present overnight ICP measurements provide a reliable picture of ICP alterations in the present patients with IIH.

### The Glia-Neuro-Vascular Interface

This present work draws attention to the glia-neuro-vascular interface in IIH. The estimated total perfused length of capillaries in the human brain is about 600–700 km, covering a total surface area of 20 m^2^ and a median inter-capillary distance of 50 μm (Winkler et al., 2014; Sweeney et al., 2016). There is one capillary for each neuron in the brain; the distance between a capillary and a neuron is about 10 μm. The astrocytes have processes that approach both the neuropil and the capillaries, the latter constituting the perivascular astrocytic end-feet that surround the entire capillary as a donut with end-feet gaps, estimated to about 20 nm toward the interstitial tissue (Mathiisen et al., 2010). The end-feet facing the basement membrane is covered by the water channel aquaporin-4 (AQP4), covering about 50% of the area of end-feet (Nagelhus and Ottersen, 2013). In adult humans, the glial cells, including the astrocytes, constitute about half of the total number of brain cells and half of the brain volume (Verkhratsky and Butt, 2013; Winters and Kleinschmidt-Demasters, 2015). The astrocytes are instrumental in bridging the functional connection between neurons and brain capillaries and play a key role in brain metabolism (Howarth, 2014). In particular, the perivascular astrocytic end-feet exhibit signaling patterns that are crucial, for e.g., vasomotion and water and fluid homeostasis (Amiry-Moghaddam and Ottersen, 2003; Mulligan and MacVicar, 2004; Boulay et al., 2017; Langer et al., 2017), and for maintenance of blood-brain barrier (BBB) function (Haddad-Tovolli et al., 2017).

A seminal study from 2012 coined the perivascular fluid circulation through brain parenchyma “the glymphatic system,” serving as a pseudo-lymphatic system within the brain (Iliff et al., 2012). The glymphatic system is a perivascular transport system for fluids and solutes, hypothesizing that waste products from cerebral metabolism are transported antegrade at the arterial side of the capillary network, *via* interstitial tissue, with efflux antegrade along the venous side of the capillaries. The system depends on AQP4 at the astrocytic end-feet, is regulated by sleep, and becomes impaired with increasing age (Nedergaard and Goldman, 2020). We recently provided evidence of impaired glymphatic function in patients with IIH (Eide et al., 2021b).

**Figure 12:**
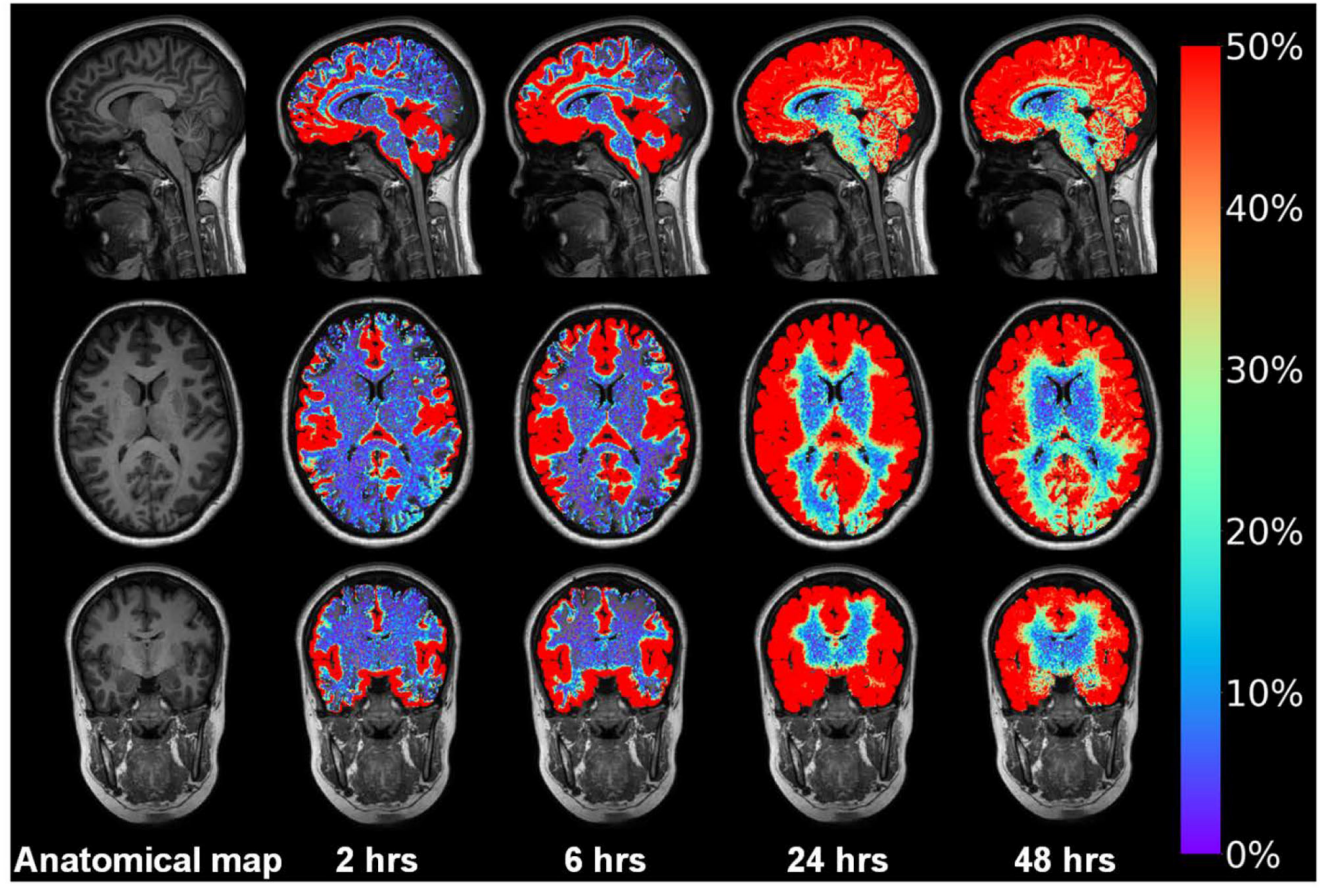
Intrathecal contrast-enhanced MRI in one subject with IIH provides evidence for a glymphatic system in patients with IIH. Intrathecal injection of the MRI contrast agent gadobutrol (Gadovist^tm^, Bayer Pharma AG, Berlin, Germany) in a dose 0.5 mmol, serves as a CSF tracer molecule. Gadobutrol has a molecular size of about 604 Da and is highly hydrophilic, making it suitable for transport within the CSF. After arriving to the subarachnoid CSF, the tracer is contained outside the blood vessels since it does not pass the BBB. Using FreeSurfer, tracer in CSF spaces is subtracted, only showing enrichment of tracer in brain parenchyma over time in this IIH patient responding to shunt surgery. The tracer enriched in the interhemispheric fissure and Sylvian fissure and medial temporal where the large vessels resides anterior middle cerebral and posterior cerebral arteries, respectively. Moreover, the tracer enriched in a centripetal fashion; at 24 h the entire brain had enriched with tracer that lasted at 48 h. *In vivo* evidence for impaired glymphatic function in IIH has been given before (Eide et al., 2021b). Illustration: Lars Magnus Valnes, Department of neurosurgery, Oslo university hospital, Oslo, Norway.

Figure 12 demonstrates in a shunt-responsive IIH patient the extravascular enrichment within brain parenchyma of an MRI contrast agent (gadobutrol, molecular size 604 Da), serving as CSF tracer after injection to the lumbar subarachnoid space. This tracer is hydrophilic and does not pass the BBB and is transported outside the capillaries. Hence, the abnormal extravascular passage of this CSF tracer previously shown in patients with IIH (Eide et al., 2021b) should draw our attention toward the glia-neuro-vascular interface, wherein the basement membrane represents a loose matrix for solute transport, and wherein the function of astrocytic end-feet would be expected to affect molecular transport *via* inter-end-feet gaps and along the capillaries.

### IIH Is Characterized by Alterations at the Glia-Neuro-Vascular Interface

In this study, ultrastructural changes at the glia-neuro-vascular interface were observed in 12/13 (92%) of IIH subjects, despite no imaging signs of sinus vein stenosis, and overnight mean ICP of >15 mmHg in merely 3/13 (23%) of IIH subjects. On the other hand, abnormal pulsatile ICP was seen in 13/13 of patients with IIH.

The present IIH subjects were shunt responders and demonstrated patchy astrogliosis in cortical gray matter, including cell hypertrophy, and loss of astrocyte domains, which compares with previous observation in non-selected patients with IIH treated surgically or conservatively (Eide et al., 2016). Astrogliosis was visualized by increased expression of the glial fibrillary acidic protein (GFAP). In general, an astrogliosis is a non-specific event of astrocytes, which may occur secondary to a wide range of causes, such as noxious agents, chemokines, cytokines, trauma, infections, and inflammations (Oberheim et al., 2012; Verkhratsky and Butt, 2013; Winters and Kleinschmidt-Demasters, 2015). Astrogliosis also affects the end-feet processes and their molecular composition (Eid et al., 2005; Heuser et al., 2012).

An important observation in the patients with IIH is that the perivascular expression of AQP4 was not reduced, as opposed to the dementia subtype idiopathic normal pressure hydrocephalus (iNPH), which presented with loss of perivascular AQP4 expression (Eide and Hansson, 2018; Hasan-Olive et al., 2019a). It is, therefore, of note that *in vivo* evidence suggests impaired glymphatic function in IIH (Eide et al., 2021b), even though perivascular AQP4 expression was not reduced. On the contrary, in IIH subjects the degree of perivascular AQP4 expression correlated positively with the degree of astrogliosis, i.e., an increasing degree of astrogliosis was accompanied with an increasing degree of AQP4 expression (Eide et al., 2016). This may be interpreted as indicative of a compensatory increase of AQP4 at perivascular astrocytic end-feet.

In the astrocytic processes close to the perivascular end-foot membrane, 3-dimensional (3D) EM showed bundles of mitochondria (Mathiisen et al., 2010). We used transmission electron microscopy (TEM) to study mitochondria in astrocytes and found an increased frequency of pathological mitochondria within the perivascular astrocytic end-feet of IIH subjects (Eide et al., 2021a). Furthermore, the degree of astrogliosis correlated positively with a number of pathological mitochondria in duplicate samples from the same patient; i.e., more astrogliosis and more pathological mitochondria. It has previously been shown that mitochondrial dysfunction is associated with reactive cortical astrogliosis (Fiebig et al., 2019).

Regarding neuronal dysfunction in IIH, in the present IIH shunt responders, there were shortened mitochondria-endoplasmic reticulum contact (MERC) distance in neuronal soma and shortened post-synaptic density (PSD) length. The role of MERCs is complex (Giacomello and Pellegrini, 2016). We interpret our observations of increased occurrence of pathological structures in neuronal soma of patients with IIH as indicative of abnormal cellular metabolism in neurons (Eide et al., 2021a). Comparable observations were previously made in individuals with the neurodegenerative disease and dementia subtype idiopathic normal pressure hydrocephalus (Leal et al., 2018; Hasan-Olive et al., 2019b). Moreover, the observation of reduced post-synaptic density length in IIH shunt responders is suggestive of impaired synaptic strength. This observation is of interest, given reports of cognitive impairment being prevalent in patients with IIH (Kharkar et al., 2011; Yri et al., 2014). In line with this, we previously reported an increased frequency of pathological mitochondria in pre- and post-synaptic nerve terminals, indicative of impaired mitochondrial trafficking (Eide et al., 2021a). Due to the high-energy demand in the pre- and post-synaptic terminals, a sufficient number of functional mitochondria is a prerequisite to maintaining synaptic function (Hollenbeck, 2005; Yu and Yu, 2017). Another piece of evidence of neuronal dysfunction was the finding of synaptic stripping in 5/13 (54%) of patients with IIH. The phenomenon of synaptic stripping is a process by which microglia selectively remove synapses from injured neurons (Blinzinger and Kreutzberg, 1968). Taken together, there is accumulating evidence for neuronal dysfunction in IIH disease.

The present observations confirmed structural changes in capillaries of patients with IIH responding to shunt surgery. Capillary damage was indicated by the frequent occurrence of caveolae and endothelial cytoplasmic vesicles, with the basement membrane being folded, tortuous, and irregular with signs of splitting, as previously shown in non-selected patients with IIH, treated conservatively or surgically (Eidsvaag et al., 2018). Of note, the pericyte processes in IIH were degenerating despite structurally intact pericyte cell bodies. It has previously been shown that intact pericytes are crucial for the development, establishment, and maintenance of the BBB (Liwnicz et al., 1990; Farkas and Luiten, 2001; Sagare et al., 2013; Andreone et al., 2017; Erdo et al., 2017; Kisler et al., 2017; Montagne et al., 2017; Liebner et al., 2018; Sweeney et al., 2018). Experimental studies show that pericyte dysfunction causes neurovascular and metabolic dysfunction (Kisler et al., 2017) and enhances neurodegeneration (Sagare et al., 2013; Sweeney et al., 2016). Furthermore, loss and degeneration of pericytes may result in BBB dysfunction and disturbed cerebral microcirculation (van Vliet et al., 2015; Sweeney et al., 2018). In this regard, it is of interest to note the occurrence of leakage of the blood glycoprotein fibrin (ogen) in IIH shunt responders, as well as in patients with IIH treated surgically or conservatively (Hasan-Olive et al., 2019c). Fibrin (ogen) has a molecular weight of 340 kDa and is a marker of BBB integrity. Outside the blood vessels, fibrinogen is transformed into fibrin. These blood products are pro-inflammatory, and extravasated fibrin (ogen) promotes inflammation (Alafuzoff et al., 1985; Paul et al., 2007; Zlokovic, 2008; Sengillo et al., 2013; Sweeney et al., 2016; Liebner et al., 2018). There was a positive correlation between the degree of the astrogliosis and degree of BBB leakage (Hasan-Olive et al., 2019c). Considering the microvascular changes seen in IIH, it should be noted that patients with IIH have a substantially increased cardiovascular risk (Fric et al., 2017; Adderley et al., 2019), and female patients a with IIH have higher prevalence of diabetes and arterial hypertension as compared to the general population (Fric et al., 2017).

Some limitations regarding comparisons between IIH and REF subjects should be noted. Gender differences between groups might play a minor role (female/male ratio 11/2 vs. 7/5). The IIH and REF groups also differed concerning BMI. Differences regarding the location of brain biopsy and minor differences in the layers assessed might as well play a role. Whether or not long-term treatment with acetazolamide or topiramate for IIH cause histological changes is to our knowledge unknown. In the epilepsy REF subjects, long-term treatment with anticonvulsants might have affected the histochemical findings. Abnormal changes in REF samples would rather be expected to reduced differences toward IIH. Even though our REF subjects were not healthy in all respects, the tissues samples were gathered from brain not affected by the primary diseases, but that anyway had to be removed as part of treatment.

### Role of Neuro-Inflammation in IIH

While BBB leakage of blood proteins are pro-inflammatory and the patchy astrogliosis of IIH may be caused by inflammation, this study revealed no increased CD68 expression. CD68 is an indicator of microglia activation (Eide et al., 2016). Microglia is a glial cell type that may become activated in neurodegenerative diseases. Hence, serum and CSF of IIH subjects showed pro-inflammatory cytokines, including interleukin (IL)-1β, IL-8, and tumor necrosis factor (TNF)-α (Edwards et al., 2013; Samanci et al., 2017). In this context, the recent observations of anti-glial autoimmunity may have relevance. In multiple sclerosis patients, the immuno-modulating treatment daclizumab resulted in secondary GFAP autoimmunity (Luessi et al., 2018). A GFAP-antibody positive meningo-encephalitis is a steroid-responsive encephalitis that may be accompanied with meningitis, myelitis, and optic disc edema and evidence of inflammation in CSF (Fang et al., 2016; Flanagan et al., 2017; Shan et al., 2018). Some patients with this entity share similarities with IIH, such as visual impairment, papilledema, visual field defects, and intracranial hypertension (Flanagan et al., 2017; Chen et al., 2018). A subset of patients with IIH were positive toward GFAP-antibody, which might in some IIH subjects result in astrocyte dysfunction (Yetimler et al., 2021). Therefore, further focus on neuro-inflammation in IIH is warranted.

### New Perspectives on the Pathophysiology of IIH

The authors suggest that the present observations provide new perspectives on the pathophysiology of IIH. In a possible cascade, capillary damage and BBB disruption may be primary events, possibly related to overweight, medication, or even genetic factors. Leakage of blood products, such as fibrin and fibrinogen, may induce inflammatory responses and astrogliosis that may affect capillary-mediated energy supply and peri-capillary clearance of metabolic waste causing energy failure and altered metabolism in astrocytes and neurons. Notably, astrogliosis in Patients with IIH may as well be triggered by obesity (Horvath et al., 2010; Hao et al., 2016). Altered BBB function and astrogliosis may in turn increase brain volume and impair the intracranial pressure-volume reserve (i.e., intracranial compliance) and even increase the ICP. In this model, venous compression and increased CSF pressure are secondary events.

In particular, the astrogliosis may be crucial for the cranial nerve dysfunction that characterizes IIH. Previously, the optic nerve path between the chiasm and the eye globe in adult humans was mapped (Hayreh, 1984) and demonstrated the tight-fitting passage of the optic nerve through the bony optic canal, a location where the nerve is susceptible to compression and distortion. Impairment of the glial cells that support the function of the optic nerve axons leads to axonal and eventually nerve cell body degeneration (Lee et al., 2012). The observed astrogliosis in IIH may therefore be accompanied with reduced glial metabolic support to the optic nerve, which may contribute to the impairment of visual function. We found in patients with IIH with visual impairment significantly higher GFAP immunoreactivity (and perivascular AQP4 expression) than in those without visual impairment (Eide et al., 2016). Furthermore, the increased optic nerve volume and subarachnoid CSF accumulation (Alperin et al., 2013) may be relieved by surgical fenestration of the optic sheath, which in some patients with IIH counteract the visual impairment (Feldon, 2007). Since all cranial nerves escape the cranial vault *via* openings in the scull base, minor volume increase might underlie the cranial nerve dysfunction characterizing IIH, e.g., visual failure, reduced olfactory function, tinnitus, and hearing loss (Jindal et al., 2009; Schmidt et al., 2012; Kunte et al., 2013; Bershad et al., 2014; Reitsma et al., 2015; Mollan et al., 2016).

With regard to the abnormal pulsatile ICP, characterizing IIH (Eide, 2021), dysfunction of the perivascular astrocytic end-feet may be crucial. Even though the water channel AQP4 covers about 50% of the astrocytic end-feet facing the basement membrane, the exact role of the AQP4 channel is unknown. Tentatively, it may be suggested that AQP4 enables variable influx of water to the end-feet processes, thereby regulating the volume and structure of the end-foot, which secondarily regulates the size of the inter-end-feet gaps. In turn, this enables variable efflux of molecules from the basement membrane to the interstitial space. Volume regulation of donut-shaped perivascular astrocytic end-feet may also enable a cushioning effect of the pulsatile pressure changes created by the capillary pulsations at the arterial (and venous) side. Hypothetically, dysfunctional astrocytic end-feet forming a donut around the capillaries may be a less efficient pressure pulsation absorber, thereby, explaining the elevated pulsatile ICP in patients with IIH. Furthermore, dysfunctional astrocytic end-feet may be a biological cause of the impaired pulsation absorber mechanisms and abnormal pulsatile ICP seen in other diseases, such as idiopathic normal pressure hydrocephalus (Park et al., 2012).

## Conclusion

Our knowledge about IIH has evolved since its first description in 1937, with current pathophysiological thinking focusing primarily on the intracranial CSF and venous pressures. The results presented here suggest that events occurring at the glia-neuro-vascular interface are important players in IIH pathophysiology. The role of sinus vein stenosis is questionable. Understanding the underlying molecular events might pave the way toward more specific treatment of the IIH disease.

## Data Availability Statement

The original contributions presented in the study are included in the article/supplementary material, further inquiries can be directed to the corresponding author.

## Ethics Statement

The studies involving human participants were reviewed and approved by the Regional Committee for Medical and Health Research Ethics of Health Region South-East, Norway (Approvals no. REK 2009/2060, 2012/1157, and 2011/2306). The patients/participants provided their written informed consent to participate in this study.

## Author Contributions

PE: conceptualization, design, writing—original draft, supervision, administration, correspondence, and material requests. PE and H-AH: data analysis, review, editing, and approval of the final manuscript. Both authors contributed to the article and approved the submitted version.

## Funding

The work involving histopathological assessment of brain tissue specimens was supported by grants from Health South-East, Norway (Grant Nos. 2012016 and 2016027).

## Conflict of Interest

Figure 3 is from Sensometrics analytical software, which is used for digital recording of continuous pressure (ICP, ABP, and CSF pressure) signals. PE has a financial interest in the software company, dPCom AS, which manufactures this software. The remaining author declares that the research was conducted in the absence of any commercial or financial relationships that could be construed as a potential conflict of interest.

## Publisher's Note

All claims expressed in this article are solely those of the authors and do not necessarily represent those of their affiliated organizations, or those of the publisher, the editors and the reviewers. Any product that may be evaluated in this article, or claim that may be made by its manufacturer, is not guaranteed or endorsed by the publisher.

## Abbreviations

A: astrocyte
AQP4: aquaporin-4
BBB: blood-brain-barrier
BIH: benign intracranial hypertension
BM: basement membrane
BMI: body mass index
CD68: Cluster of differentiation 68
CSF: cerebrospinal fluid
CRP: C-reactive protein
EC: endothelial cell
EF: endfoot
ER: endoplasmic reticulum
GFAP: glial fibrillary acidic protein
ICP: intracranial pressure
IIH: Idiopathic intracranial hypertension
IL: interleukin
iNPH: idiopathic normal pressure hydrocephalus
LM: light microscopy
LP: lumboperitoneal
M: mitochondria
MERC: mitochondria-endoplasmic reticulum contact
MRI: magnetic resonance imaging
MWA: mean ICP wave amplitude
N: neuron
NF-H: neurofilament-heavy
P: pericytes
Pp: pericyte process
PSD: postsynaptic density
PTC: pseudo-tumor cerebri
REF: reference
TEM: transmission electron microscopy
TNF: tumor necrosis factor
VP: ventriculoperitoneal.
